# Comparative Review of *O*,*O*′-, *N*,*O*-, and *N*,*N*′-Bidentate Ligands: Structural and Electronic Properties of β-Diketones, Enaminones, and β-Diketiminates

**DOI:** 10.3390/molecules31071223

**Published:** 2026-04-07

**Authors:** Jeanet Conradie

**Affiliations:** Department of Chemistry, University of the Free State, P.O. Box 339, Bloemfontein 9300, South Africa; conradj@ufs.ac.za

**Keywords:** β-diketones, imino-β-diketones, enaminones, β-diketiminates, bidentate ligands, tautomerism, density functional theory, frontier molecular orbitals, electrochemistry, structure–property relationships

## Abstract

Bidentate ligands, derived from the 1,3-dicarbonyl framework, play a central role in coordination chemistry, catalysis, and materials science due to their tuneable donor properties and structural versatility. This review examines and compares three closely related ligand classes, β-diketones (O,O′ donors), imino-β-diketones or enaminones (N,O donors), and di-imino-β-diketones or β-diketiminates (N,N′ donors), to elucidate how systematic substitution of oxygen by nitrogen affects structure and properties. The discussion integrates spectroscopic data (NMR and IR), crystallographic findings, electrochemical measurements, and density functional theory (DFT) calculations reported in the literature. Across these systems, tautomerism plays a decisive role, with conjugation-stabilized enol or enamine forms generally preferred in solution and the solid state. Frontier molecular orbital analyses show extensive delocalization over the chelate backbone and, when present, aromatic substituents. Electrochemical studies reveal consistent correlations between experimental reduction potentials and calculated LUMO energies for *O*,*O*′-, *N*,*O*-, and *N*,*N*′-bidentate ligands. Overall, the comparison demonstrates that donor atom substitution within a conserved conjugated scaffold provides a systematic approach to tuning acidity, coordination behaviour, and redox properties, offering a coherent framework for understanding structure–property relationships in 1,3-dicarbonyl-derived chelating ligands.

## 1. Introduction

Over the past several decades, the chemistry of transition metal complexes has expanded tremendously, driven by the synthesis of new ligands, the discovery of novel coordination modes, and the development of advanced structural and spectroscopic characterization techniques. Despite the continuous emergence of sophisticated ligand frameworks, a number of classical ligands that have been known for many decades remain central to coordination chemistry, due to their predictable binding modes, tuneable steric and electronic properties, and versatile applications in catalysis, materials science, and bioinorganic chemistry.

Among the earliest and most extensively studied ligands are *O*,*O*′- and *N*,*N*′-chelating systems. Classical examples include β-diketones, such as acetylacetone (2,4-pentanedione); salicylaldehyde-derived Schiff bases; 1,2-diamines, such as ethylenediamine; and aromatic diimines, such as 2,2′-bipyridine and 1,10-phenanthroline. These ligands coordinate metals through nitrogen and/or oxygen donor atoms, forming five- or six-membered chelate rings that confer enhanced thermodynamic stability via the chelate effect. Their modular structures allow systematic modification of steric bulk and electronic properties, making them ideal platforms for structure–property investigations.

**Scheme 1:**
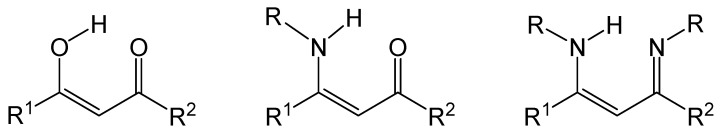
Structures of O,O′-, N,O-, and N,N′-bidentate ligands in this work: β-diketones, imino-β-diketones (enaminones) and di-imino-β-diketones (β-diketiminates); only one enol form shown.

This review focuses on a selected group of structurally related bidentate ligands that coordinate through O,O′, N,O, or N,N′ donor sets and that share a common β-dicarbonyl-type backbone or closely related conjugated frameworks; see Scheme 1.

β-Diketones (O,O′ ligands), with the general structure R^1^–C(O)–CH_2_–C(O)–R^2^ in the keto form, are prototypical O,O′-chelating ligands. They exist in equilibrium with the enol tautomer R^1^–C(O)–CH=C(OH)–R^2^, the latter often stabilized by intramolecular hydrogen bonding and conjugation. Upon deprotonation, β-diketones form resonance-stabilized enolate anions that bind metal ions through both oxygen atoms, generating six-membered chelate rings. Their strong π-delocalization, tuneable substituents (R, R′), and well-defined coordination behaviour have made them fundamental ligands in coordination chemistry, from classical metal acetylacetonates to advanced functional materials [1].

Replacement of one carbonyl oxygen by an imino group leads to α,β-unsaturated ketones or imino-β-diketones (N,O ligands) with general structure R^1^–C(O)–CH_2_–C(NR)–R^2^ (keto form). These ligands introduce an N,O donor set while retaining conjugation across the backbone. The presence of the imine nitrogen modifies both the donor strength and the electronic distribution within the chelate ring. Tautomerism, conjugation, and substituent effects play decisive roles in determining their coordination modes, acidity, and redox properties [2]. Compared to purely O,O′-donor β-diketones, N,O systems provide enhanced opportunities for electronic fine-tuning and metal–ligand π-interactions.

Further substitution of both carbonyl functionalities by imine groups generates di-imino-β-diketone-type ligands (N,N′ ligands) with general structure R^1^–C(NR)–CH_2_–C(NR′)–R^2^ (keto form). These N,N′-chelating systems combine the structural motif of β-diketones with the coordination characteristics of diimines. The resulting ligands typically exhibit extended π-conjugation and strong σ-donor/π-acceptor capabilities, depending on the substituents at nitrogen. Such frameworks bridge the chemistry of classical β-dicarbonyls and aromatic diimines, offering a versatile platform for modulating metal-centred redox processes and electronic structures [3,4,5]. Understanding the structure and properties of O,O′-, N,O-, and N,N′-bidentate ligands is essential for rationalizing and predicting the behaviour of their metal complexes.

This review provides a systematic comparative analysis of β-diketones, imino-β-diketones, and β-diketiminates, focusing on how stepwise substitution of oxygen donor atoms by nitrogen fundamentally alters ligand electronics, steric profiles, and coordination behaviour. By integrating structural data, electrochemical trends, and computational descriptors, it is shown that these ligand classes follow consistent and predictable structure–property relationships, particularly in terms of frontier orbital energies and redox potentials. Importantly, this comparison reveals trends that are not apparent when these systems are considered in isolation, including the progressive impact of donor substitution on metal–ligand bonding, redox non-innocence, and coordination number limitations. For example, increasing steric and electronic demands across the series rationalize the reduced occurrence of higher-coordinate species such as M(IV)(enaminone)_4_ and M(IV)(β-diketiminate)_4_ compared to the well-established M(IV)(β-diketonato)_4_ complexes. This unified perspective provides a framework for the rational design of ligands with tailored properties.

## 2. Discussion

The following section examines three closely related classes of bidentate ligands derived from the 1,3-dicarbonyl framework, highlighting how systematic donor atom substitution influences their structural and electronic properties. Particular emphasis is placed on comparing classical β-diketones (O,O′ donors) with their nitrogen-containing analogues, imino-β-diketones (N,O donors) and β-diketiminates (N,N′ donors), to establish clear structure–property relationships across the series.

### 2.1. Beta-Diketones (O,O′-Ligands)

β-Diketones (1,3-disubstituted propane-1,3-diones) are among the most extensively investigated O,O′-chelating ligands in coordination chemistry. For more than a century, their metal complexes have been studied in detail, and their structural diversity and rich coordination behaviour are well documented [1]. β-Diketonato complexes are known for a wide range of transition metals [6,7,8], and they continue to serve as benchmark systems in catalysis, materials chemistry, and precursor design.

The general structure of a β-diketone in its neutral keto form is R^1^–C(O)–CH_2_–C(O)–R^2^. Variation of the substituents R^1^ and R^2^ provides powerful control over steric and electronic properties. Frequently used examples include acetylacetone (Hacac), hexafluoroacetylacetone (Hhfaa), dibenzoylmethane (Hdbm), benzoyltrifluoroacetone (Htfba), and trifluoroacetylacetone (Htfaa). Electron-withdrawing substituents (e.g., CF_3_) enhance acidity and metal-binding strength, while aromatic substituents extend conjugation and influence redox and spectroscopic properties. This synthetic flexibility has resulted in a vast library of β-diketones tailored for specific coordination and functional applications.

A defining feature of β-diketones is their ability to form stable six-membered chelate rings upon deprotonation of the central methylene group, generating a resonance-stabilized β-diketonate anion. The combination of strong σ-donation and π-delocalization underpins the thermodynamic stability of their metal complexes and makes them ideal systems for systematic structure–property investigations.

#### 2.1.1. Tautomerism

**Scheme 2:**
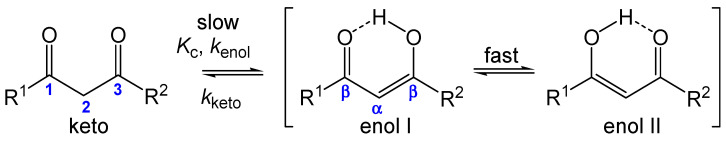
Keto and enol tautomers of β-diketones, including the numbering of the backbone carbons in blue numbers. The enol–enol tautomerization is fast and the keto–enol tautomerization slow, with equilibrium constant *K*_c_.

Tautomerism is central to the chemistry of β-diketones because it directly determines their structure, acidity, and coordination behaviour. In solution, β-diketones exist in equilibrium between a diketo form and two symmetry-related enol forms (enol I and enol II; see Scheme 2). The enol forms arise from proton transfer from the central methylene carbon to one of the carbonyl oxygen atoms, accompanied by formation of a conjugated O–C=C–C=O π-system.

##### Keto–Enol Equilibrium

In most non-polar and aprotic solvents, the enol form predominates; typically exceeding 80% at room temperature [1]. Its stability arises from two synergistic effects, namely from intramolecular hydrogen bonding, forming a six-membered pseudo-chelate ring (O–H···O), and from π-electron delocalization across the conjugated backbone [9]. The enol content generally decreases with increasing temperature due to weakening of the intramolecular hydrogen bond. Solvent polarity also plays a decisive role: polar protic solvents may stabilize the diketo form through intermolecular hydrogen bonding, whereas non-polar solvents favour the internally hydrogen-bonded enol [10].

Substituent effects strongly influence the equilibrium. Electron-withdrawing groups (e.g., CF_3_) increase enol content by stabilizing the conjugated system and enhancing acidity of the central proton [11]. Aromatic substituents similarly promote enolization through extended conjugation. In contrast, bulky alkyl groups at the α-position may reduce enol content through steric repulsion and inductive effects.

##### Enol–Enol Tautomerism and Driving Forces

For unsymmetrical β-diketones (R^1^ ≠ R^2^), two enol isomers are possible (Scheme 2). Their relative abundance is governed by two principal factors, the electronic (inductive) effects, related to the electronegativity of R^1^ and R^2^, and resonance stabilization, particularly when aromatic substituents are present [12,13].

If one substituent is significantly more electron-withdrawing, the preferred enol form according to the electronic driving force is typically adjacent to the more electron-donating group. However, numerous exceptions, especially in systems bearing aromatic or ferrocenyl substituents, demonstrate that resonance effects often override simple electronegativity arguments. In such cases, the enol isomer that allows greater π-delocalization through aromatic conjugation is preferentially stabilized [12,13].

Spectroscopic and crystallographic studies provide indirect evidence for this resonance-driven preference, as reflected in bond lengths within the chelated enol fragment [14,15].

The interconversion between the two enol isomers is extremely rapid (rate constants approaching 10^6^ s^−1^), typically fast on the NMR timescale, resulting in averaged spectroscopic signals [16]. Advanced techniques, such as ^17^O and ^13^C NMR spectroscopy [16,17] and quantum chemical calculations [18], have been employed to estimate enol–enol equilibrium constants, highlighting the sensitivity of the equilibrium to substituent identity and position.

##### Kinetics of Keto–Enol Interconversion

For most simple β-diketones (e.g., Hacac, Hhfaa, Htta), keto–enol interconversion is rapid and solvent-dependent [19]. However, in certain systems, such as cyclic β-diketones (e.g., 2-acetylcyclohexanone [19]) and some ferrocenyl-substituted derivatives, the tautomerization is sufficiently slow to be monitored by conventional spectroscopic methods (UV/Vis or ^1^H NMR) [12,13,20]. Reported half-lives can extend to several hours.

Kinetic studies have demonstrated that sample history and preparation can significantly affect observed tautomer ratios. For example, β-diketones isolated via their lithium salts are initially obtained in the diketo form; subsequent conversion to the thermodynamically favoured enol form in solution may proceed slowly. Consequently, freshly prepared samples may show unusually high keto content, whereas aged samples approach equilibrium distributions. In the solid state, the enol form is generally dominant due to stabilization by intramolecular hydrogen bonding and crystal packing effects [12,13].

The equilibrium constant (*K*_c_) for keto–enol tautomerism is defined by the ratio of forward and reverse rate constants and is strongly influenced by solvent, substituents, and temperature. Quantitative evaluation has been achieved using NMR integration [12,13], UV/Vis spectrophotometry [20], isotopic exchange experiments, and computational methods [21].

#### 2.1.2. Protonation States and Acidity (pK_a_ of β-Diketones)

The acidity of β-diketones is a fundamental parameter governing their coordination chemistry. The p*K*_a_ of a β-diketone refers to the equilibrium constant for deprotonation of the central methylene proton in aqueous solution:

R^1^–CO–CH_2_–CO–R^2^ ⇌ R^1^–CO–CH^−^–CO–R^2^ + H^+^

**Scheme 3:**
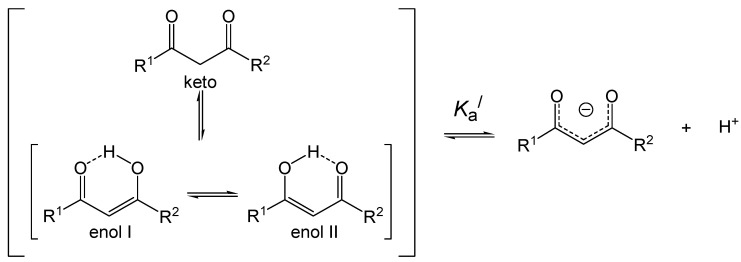
Deprotonation reaction that occurs during the determination of p*K*_a_^/^values of β-diketones.

In practice, the experimentally determined value corresponds to the acidity of the tautomeric mixture present in solution. Because keto and enol forms coexist and interconvert, the measured quantity is often referred to as an apparent p*K*_a_ (p*K*_a_′) [22]; see Scheme 3. This value cannot usually be partitioned into separate p*K*_a_ values for the individual keto and enol tautomers, since deprotonation and tautomerization are thermodynamically and kinetically coupled processes.

The enhanced acidity of β-diketones compared to simple ketones arises from stabilization of the conjugate base (β-diketonate anion). Upon deprotonation, the negative charge is delocalized over both carbonyl oxygen atoms through resonance, forming a planar, π-conjugated O–C=C–C=O system. The same electronic delocalization that stabilizes the enol tautomer also stabilizes the anion, thereby lowering the p*K*_a_ and facilitating chelate formation with metal ions.

Electron-withdrawing substituents on R^1^ and/or R^2^ further stabilize the conjugate base via inductive and resonance effects, significantly lowering the p*K*_a_. Conversely, electron-donating substituents destabilize the anion and increase the p*K*_a_.

Several experimental techniques are used to determine the p*K*_a_′ values of β-diketones, such as potentiometry [23], based on pH titration curves [22], conductometry [24], monitoring conductivity changes during deprotonation and different spectroscopic methods, including UV/Vis [23,25], vibrational (IR) [26,27], and ^1^H NMR [26] spectroscopy.

Among these, UV/Vis spectrophotometry is particularly convenient because the neutral β-diketone and its conjugate base typically exhibit distinct absorption bands and extinction coefficients. Monitoring absorbance as a function of pH allows accurate determination of the apparent p*K*_a_. NMR spectroscopy provides complementary structural insight, especially when tautomeric equilibria must be considered.

A wide range of β-diketones has been systematically investigated, revealing a broad acidity range spanning nearly nine p*K*_a_ units. Representative compounds ordered by increasing p*K*_a_ (i.e., decreasing acidity) include the following [12,22,28,29,30] (see List of Abbreviations for β-diketone names):

Hhfaa (4.71) < Htfba (6.3) ≈ Htfaa (6.3) < Htta (6.49) < Hfctfa (6.56) < Htfhd (6.64) < Htfdma (6.80) < Hfch (7.04) < Hfctca (7.13) ≈ Htftma (7.13) < Hba (8.70) < Hdtm (8.89) < Hacac (8.95) < Hbth (9.01) < Hdbm (9.35) < Hfca (10.01) < Hbfcm (10.41) < Hdfcm (13.1).

At the acidic end of the series, hexafluoroacetylacetone (Hhfaa), bearing two strongly electron-withdrawing CF_3_ groups, exhibits a p*K*_a_ of 4.71. The high group electronegativity of CF_3_ substituents stabilizes the anion efficiently, resulting in strong acidity. In contrast, β-diketones substituted with electron-rich ferrocenyl (Fc) groups, such as Hdfcm, show markedly higher p*K*_a_ values (up to 13.1), reflecting reduced stabilization of the conjugate base.

Because efficient electronic communication exists between both substituents and the conjugated β-diketone framework, their effects are commonly treated as additive. Consequently, as the electron withdrawing power of the substituents, as expressed by Gordy scale group electronegativities ($\chi_{R}$) or the Hammett meta substituent constants ($\sigma_{R}$), increases, the p*K*_a_ decreases. Although the relationship is not strictly linear, owing to resonance contributions, steric effects, and solvent interactions, a clear inverse correlation between substituent electron-withdrawing power and p*K*_a_ is observed (see Section 2.1.6). Aromatic substituents introduce additional resonance effects that may either enhance or moderate inductive trends, depending on their electronic nature and conjugation with the β-dicarbonyl backbone.

The p*K*_a_ of a β-diketone has relevance to coordination chemistry, since it directly determines the ease of deprotonation and formation of the chelating β-diketonate anion, the stability constants of metal complexes, competition with other ligands in solution, and redox and electronic properties of the resulting complexes, as electron-withdrawing substituents simultaneously influence ligand donor strength and metal-centred reduction potentials. Thus, systematic variation of substituents provides a rational strategy for tuning acidity and, consequently, metal–ligand interactions.

#### 2.1.3. Synthesis

As this review focuses primarily on the structural and physicochemical properties of β-diketones as O,O′-bidentate ligands, their synthesis will be addressed only briefly. Comprehensive discussions of synthetic methodologies are available in several dedicated reviews [31,32,33,34,35]. Here, only the most widely applied and synthetically versatile approach, the Claisen condensation, is outlined, together with a short overview of alternative strategies.

A variety of chemical and catalytic methodologies have been developed for the preparation of 1,3-diketones. These include classical base-mediated condensations, hydration of alkynones, decarboxylative coupling reactions, and more recent organocatalytic, metal-catalysed, or even biocatalytic protocols. In addition, α-functionalization of preformed 1,3-diketones provides access to branched β-dicarbonyl derivatives, further expanding structural diversity [32]. Despite these advances, the Claisen condensation remains the most general and widely employed method for synthesizing β-diketones, owing to its reliability, broad substrate scope, and operational simplicity [36].

##### Claisen Condensation

**Scheme 4:**
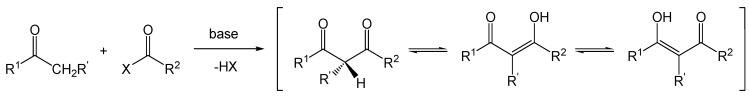
Synthesis of β-diketones by the Claisen condensation reaction: a ketone containing an α-hydrogen atom undergoes acylation with an appropriate acylation reagent (acid anhydride, an acid chloride or an ester) in the presence of a base.

The Claisen condensation involves the base-promoted acylation of a ketone containing at least one α-hydrogen with an ester (or, alternatively, an acid chloride or anhydride); see Scheme 4. In its classical form, the reaction proceeds via the following key steps [31]:

Enolate formation: A strong base (commonly alkoxides or lithium diisopropylamide, LDA) deprotonates the α-position of the ketone to generate a resonance-stabilized enolate.

Nucleophilic acyl substitution: The enolate attacks the carbonyl carbon of the ester, forming a tetrahedral intermediate that eliminates an alkoxide to yield the β-diketone framework.

Secondary deprotonation: The initially formed β-diketone is more acidic than the starting ketone and is, therefore, deprotonated under the reaction conditions to give the corresponding β-diketonate salt.

Acidic workup: Protonation during aqueous workup affords the neutral β-diketone.

The overall equilibrium is driven toward product formation by the enhanced acidity of the β-diketone and, in many cases, by precipitation of its metal salt (e.g., lithium or sodium β-diketonate). The reaction tolerates a wide range of substituents, allowing systematic variation of R^1^ and R^2^ groups, which is essential for tuning acidity, steric properties, and electronic effects in coordination chemistry.

Modified Claisen-type protocols have been developed to accommodate base-sensitive substrates, including the use of softer enolate equivalents derived from carboxylic acids or alternative acylating agents.

##### Alternative Methods

Although less universally applicable than the Claisen condensation, several complementary methods are noteworthy, such as the hydration of alkynones, providing access to 1,3-diketones via addition of water across activated carbon–carbon triple bonds; the decarboxylative coupling reactions of suitable carbonyl precursors; reactions of organometallic reagents (e.g., Grignard reagents) with β-keto acid derivatives or nitriles, followed by hydrolysis and Friedel–Crafts acylation routes, particularly for aryl-substituted β-diketones. While these strategies can be advantageous in specific cases, especially for highly substituted or aromatic systems, they are generally less straightforward or less broadly applicable than the Claisen methodology [32,37,38].

From a coordination chemistry perspective, the key advantage of β-diketone synthesis lies in the modular introduction of substituents at the R^1^ and R^2^ positions. This structural tuneability enables systematic control over acidity (p*K*_a_) and ease of deprotonation, electronic effects (inductive and resonance contributions), steric bulk and bite angle in metal complexes, and redox and spectroscopic properties. Consequently, synthetic accessibility through robust condensation chemistry has been central to the widespread use of β-diketones as prototypical O,O′-chelating ligands and as model systems for correlating substituent effects with structural and electronic properties.

#### 2.1.4. Structural Information from Experiment

A detailed understanding of the geometrical and electronic structures of β-diketones is essential for rationalizing their acidity, tautomerism, and metal coordination behaviour. Structural information can be obtained from single-crystal X-ray diffraction, solution and solid-state NMR spectroscopy, vibrational spectroscopy (IR), and quantum chemical calculations (DFT). In this section, emphasis is placed on experimental structural data; computational aspects will be discussed separately.

##### Solid-State Structures

**Scheme 5:**
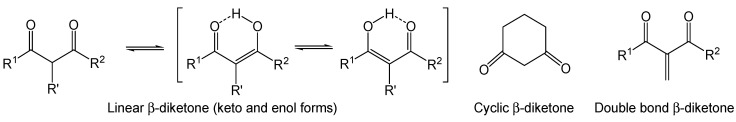
Linear, cyclic, and double-bond β-diketones.

Structurally, β-diketones may be grouped into three general categories: (i) linear 1,3-diketones, (ii) cyclic β-diketones [39,40], and (iii) conjugated systems containing additional double bonds [41]; see Scheme 5. The present discussion focuses on linear β-diketones, which constitute the most widely studied class in coordination chemistry.

Analysis of crystallographic data reveals that linear β-diketones in the solid state adopt either keto or enol forms, with the enol form being far more prevalent. A survey of the Cambridge Structural Database (CSD) [42] shows a substantially larger number of enol structures compared to diketo forms, reflecting the thermodynamic stabilization of the enol tautomer through conjugation and intramolecular hydrogen bonding.

**Figure 1:**
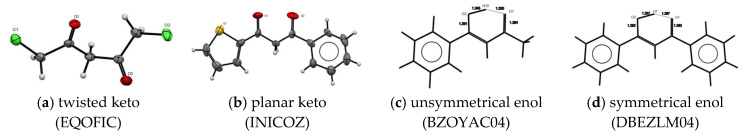
Examples of different types of published β-diketone structures (CSD [42] reference code below each structure).

In the diketo structure R^1^–C(O)–CH_2_–(C)O–R^2^, the central methylene carbon (C2) is tetrahedral. The two carbonyl groups may adopt either a twisted arrangement, with the C=O groups pointing in different directions, or a more nearly planar arrangement, depending on crystal packing and substituent effects (see examples in Figure 1). The C–C bond between the two carbonyl groups is typically a single bond (≈1.51–1.54 Å), and the C=O distances are characteristic of localized double bonds (≈1.21 Å) [15].

The most common solid-state structure is the chelated enol, characterized by a planar –C(OH)=CH–CO– backbone. A strong intramolecular O–H···O hydrogen bond forms a six-membered pseudo-ring, enforcing near-planarity and extensive π-delocalization across the O–C=C–C=O fragment [9]. In unsymmetrical β-diketones, the hydrogen bond is typically asymmetric, whereas symmetrical derivatives (R^1^ = R^2^) may exhibit nearly symmetric hydrogen bonding (Figure 1).

Characteristic bond metrics clearly reflect electron delocalization in the enol form, namely C=O (enol): elongated (≈1.27–1.28 Å), compared to the keto form, C–O (enol): shortened relative to a single bond (≈1.30–1.34 Å), C=C (central): ≈1.34–1.39 Å, and central C–C bond: shorter in enol (≈1.40–1.43 Å) than in keto (≈1.51–1.54 Å) [15]. These parameters indicate partial double-bond character distributed over the chelate fragment. The structural differences between keto and enol forms provide direct crystallographic evidence of π-electron delocalization and hydrogen bond stabilization in the enol form.

##### Structural Information from NMR Spectroscopy

NMR spectroscopy is one of the most powerful tools for investigating tautomerism and hydrogen bonding in β-diketones, both in solution and in the solid state. Because the equilibrium between diketo and enol forms is slow on the NMR timescale, signals corresponding to both species can often be observed [12,13]. In contrast, the interconversion between the two enol forms is typically fast, resulting in averaged resonances [16,17].

Several diagnostic features are particularly informative; for example, the chelate proton (enol OH) with strong intramolecular hydrogen bonding produces a highly deshielded ^1^H resonance, typically around 14–16 ppm [41]. Such downfield shifts are characteristic of low-barrier hydrogen bonds and provide qualitative information on hydrogen bond strength. The central methylene group (keto form) has a resonance near 3.0–5.5 ppm; integrating for two protons indicates the presence of the diketo tautomer, while the methine proton resonates (enol form, one proton) at lower field, generally in the range δ ≈ 5.5–7.0 ppm [12,13,41,43]. ^17^O NMR spectroscopy exploits the substantial chemical shift difference between single-bonded (C–O) and double-bonded (C=O) oxygen atoms, enabling differentiation of tautomers and evaluation of hydrogen bond effects [41]. Isotope labelling provides further insight. Partial deuteration of the enolic proton allows measurement of isotope effects on ^13^C chemical shifts, which can be used to determine the ratio between enol forms and to estimate hydrogen bond strength [44]. Both intrinsic isotope effects and equilibrium isotope effects contribute to the observed shifts, and quantitative analysis enables determination of mole fractions of individual tautomers.

Solid-state NMR has also been applied to systems with particularly strong or unusual hydrogen bonds, providing complementary structural information to diffraction methods [41].

##### Structural Information from IR Spectroscopy

Infrared spectroscopy provides a rapid and convenient method for distinguishing keto and enol forms based on characteristic carbonyl stretching frequencies. The diketo form has strong C=O stretching bands, typically appearing in the range 1680–1790 cm^−1^, depending on substitution. The enol form has conjugation and hydrogen bonding that lower the effective C=O stretching frequency. For example, the enol C=O adjacent to strongly electron-withdrawing groups (e.g., CF_3_): ~1580–1640 cm^−1^; other enol carbonyl groups: ~1650–1700 cm^−1^, and cyclic systems may exhibit slightly lower values [45].

Computational vibrational analyses have shown good agreement between calculated and experimental frequencies, allowing assignment of specific rotamers and estimation of their relative populations based on calculated free energies (ΔG). Thus, IR spectroscopy, particularly when combined with DFT calculations, is a valuable tool for probing tautomeric distributions and conformational preferences [46].

Collectively, crystallographic, NMR, and IR data consistently demonstrate that β-diketones preferentially adopt a planar, hydrogen-bonded enol structure in the solid state, whereas solution–phase equilibria may include measurable amounts of the diketo form depending on substituents, solvent, and temperature. The degree of π-delocalization, hydrogen bond strength, and substituent effects are directly reflected in bond lengths, chemical shifts, and vibrational frequencies. These structural characteristics are intimately linked to acidity, electronic distribution, and, ultimately, to metal-binding behaviour, forming the basis for understanding β-diketones as prototypical O,O′-bidentate ligands. Computational analyses of their electronic structures will be discussed in a subsequent section.

#### 2.1.5. Structural Information from DFT Studies

Density functional theory (DFT) has become an indispensable tool for investigating the structure, energetics, spectroscopy, and redox properties of β-diketones. Depending on the functional, basis set, and solvation model employed, DFT calculations can provide accurate optimized geometries, vibrational frequencies, NMR chemical shifts, frontier orbital energies (HOMO, LUMO), reduction potentials, and reaction pathways. Numerous computational studies have complemented experimental work on β-diketones, while others have focused on purely theoretical aspects of tautomerism and electronic structure.

Optimized DFT geometries of β-diketones typically show excellent agreement with single-crystal X-ray diffraction data, with root mean square deviations for non-hydrogen atoms often below 0.1 Å [47]. The characteristic features of the enol form, planarity of the O–C=C–C=O backbone and intramolecular hydrogen bonding are consistently reproduced [47]. In addition to structural parameters, DFT has been successfully applied to predict IR stretching frequencies, including differentiation between keto and enol carbonyl groups; NMR chemical shifts (^1^H, ^13^C, and in selected cases ^17^O) [41]; UV-vis spectra; and relative energies of keto and enol and tautomers [21]. Such calculations provide a theoretical framework for interpreting experimental spectroscopic data and for estimating tautomer populations.

##### Mechanism of Keto–Enol Tautomerism

**Scheme 6:**
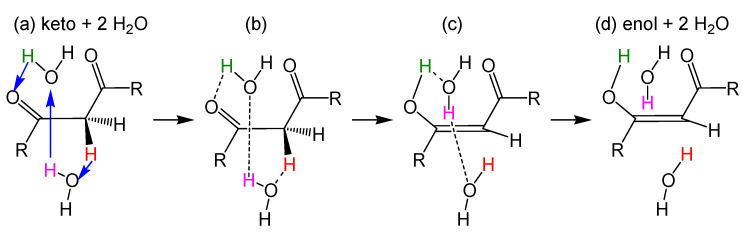
Path of keto–enol tautomerization using 2 water molecules, as proposed by Yambe [48] (**a**–**d**). The blue arrows in (**a**) indicate hydrogen bonds to be formed.

One of the earliest detailed computational analyses addressed the reaction pathway of keto–enol tautomerization. Calculations on model systems such as malonaldehyde and acetylacetone showed that a direct intramolecular proton transfer from methylene carbon to oxygen requires cleavage of a relatively strong C–H bond (≈350–390 kJ mol^−1^) and is, therefore, associated with a high activation barrier [48]. These high calculated barriers indicate that a simple, concerted intramolecular proton shift is unlikely under normal conditions. Instead, solvent-assisted proton relay mechanisms were proposed. When one or more water molecules were included explicitly in the calculations, the activation energy decreased dramatically. Hydrogen-bonded water clusters facilitate stepwise proton transfer, effectively lowering the barrier and providing a mechanistic rationale for the experimentally observed solvent dependence of tautomerization rates. See Scheme 6 for an illustration of the path of tautomerization using two water molecules, as proposed by Yambe [48].

**Figure 2:**
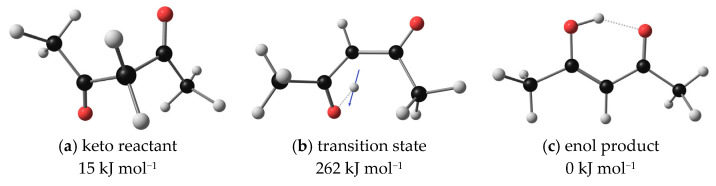
The M062X/6-311G(d,p)-optimized (**a**) keto, (**b**) transition state, and (**c**) the enol form of acetylacetone, during tautomerization. “The single imaginary frequency of the transition state (namely −2054.5 cm^−1^ with an IR intensity of 654.6 KM/mol), entails the movement of the hydrogen from C2 to O. After the transition state, rotation around the C2-C3 bond leads to the enol product state”. Relative free energies indicated. Colour code of the atoms (online version): C (grey), O (red) and H (white). “Figure reproduced from DFT reported in J. Mol. Graph. Model., 85, Adeniyi, A.A.; Conradie, J. The Stability, Kinetics and Inter-Fragment Electron Communication of the Tautomers of Twelve Selected β-Diketone Molecules: A Computational Study. 25–39, Copyright 2018, with permission from Elsevier” [21].

More recent computational studies on a broader set of 12 β-diketones typically yielded activation barriers in the range of 200–270 kJ mol^−1^ for a direct intramolecular proton transfer [21], consistent with the conclusion that solvent mediation is essential under ambient conditions. Optimized geometries further illustrate the structural transformation during tautomerization: the twisted, sp^3^-hybridized methylene carbon in the keto form becomes planar (sp^2^) in the enol form, accompanied by shortening of the central C–C bond and development of π-conjugation; see Figure 2. Solvent models (e.g., CHCl_3_, water, dioxane) and gas phase generally predict similar activation energies. The solvent media all favour the enol forms in the order of gas > dioxane > chloroform > water. Calculated equilibrium constants, *K*_c_., for the keto–enol tautomerization, i.e., the ratio of the calculated forward and reverse reaction constants (Scheme 2), correlate reasonably well with experimentally determined values in chloroform, particularly when implicit chloroform solvation is included [21].

##### Frontier Molecular Orbitals and Reduction Potentials

A major area of DFT application has been the correlation between calculated frontier orbital energies and experimentally measured electrochemical parameters. For a series of 10 substituted β-diketones, cathodic peak potentials (*E*_pc_) measured by cyclic voltammetry in acetonitrile were correlated with calculated electronic descriptors, such as LUMO energy (*E*_LUMO_), electron affinity (EA), electrophilicity index (ω), and group electronegativity parameters [47]. Excellent linear correlations were observed between experimental reduction potentials and calculated LUMO energies (R^2^ > 0.99), demonstrating that *E*_LUMO_ is a reliable predictor of reduction behaviour within homologous series. Both OLYP generalized gradient approximation (GGA) and the B3LYP hybrid functional yielded comparable trends in gas phase and implicit solvent models [47].

**Figure 3:**
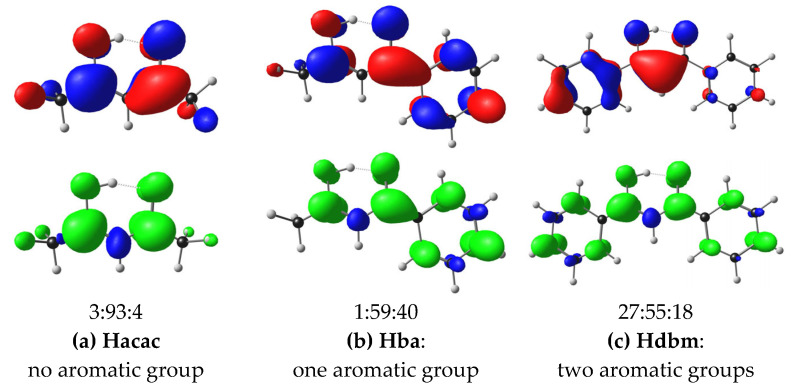
B3LYP/6-311G(d,p) LUMOs (top) of the indicated neutral β-diketones and spin density plots (bottom) of the reduced β-diketones. The % spin distribution R^1^:(C(OH)CHCO):R^2^ is shown below the spin plot. Contours of 0.05 and 0.005 e/Å^3^ were used for the MO and spin plots, respectively. Adapted from [49] (open access).

Orbital and spin density analyses of the radical anions revealed that experimentally observed reversible electrochemical behaviour is associated with effective delocalization of the added electron over a π-conjugated framework; see Figure 3. In particular, β-diketones bearing two aromatic substituents in the 1,3-positions stabilize the radical anion more efficiently by extended delocalization of the spin density over the β-diketone O–C=C–C=O backbone onto the aromatic groups, resulting in quasi- or reversible cyclic voltammograms. In contrast, systems containing only one or no aromatic substituents often exhibit irreversible reduction, due to reduced stabilization of the radical intermediate (see Electrochemistry Section 2.1.7). The stabilization of the reduced radical anion, related to aromatic groups, is illustrated by the spin density plots of the β-diketones in Figure 3. Subsequent studies extending the compound set to 15 confirmed the robustness of the linear relationship between experimental reduction potentials and calculated LUMO energies, underscoring the predictive value of DFT-derived descriptors [49].

##### Oxidation Potentials and HOMO Energies

DFT has also been employed to model anodic oxidation processes at the B3LYP/6-311+G** level. For three selected β-diketones and three β-diketoesters, experimentally measured anodic peak potentials (*E*_pa_) were correlated with calculated HOMO energies. A linear relationship between *E*_pa_ and *E*_HOMO_ was obtained, indicating that oxidation involves electron removal from orbitals of similar character across the series [50]. Such correlations provide a practical predictive tool: once calibrated for a given functional and solvent model, calculated HOMO energies can be used to estimate oxidation potentials of new β-diketone derivatives.

##### Computational Studies of pK_a_ and Redox–Acidity Relationships

More recent computational investigations have simultaneously addressed reduction potentials and p*K*_a_ values of β-diketone tautomers [51]. High-level composite methods and modern DFT functionals reproduced experimental reduction potentials with small mean absolute deviations: 0.037 eV when using the higher computational G3(MP2) method, and a slightly higher MAD of 0.061 eV when using the M06/6-311+G(2df,p) DFT method. It was found that the enol form is generally easier to reduce than the keto form, exhibiting less negative (i.e., higher) calculated reduction potentials. Within enol tautomers, enolization at the position further from a strongly electron-withdrawing substituent may lead to even higher reduction potentials [51].

The calculated deprotonation energies support the experimentally observed acidity trends and suggest that deprotonation may proceed preferentially from the enol hydroxyl group, rather than directly from the diketo form. Furthermore, calculated hydrogen bond strengths in the enol forms correlate with experimental p*K*_a_ values, reinforcing the link among intramolecular hydrogen bonding, electron delocalization, and acidity [51].

Collectively, DFT studies have clarified the mechanism and energetics of keto–enol tautomerism; reproduced experimentally observed geometrical parameters; provided quantitative correlations between frontier orbital energies and redox potentials; and offered insight into substituent effects on acidity and radical stability. These computational insights not only complement experimental structural and electrochemical data, but also enable predictive design of β-diketone ligands with tailored electronic properties. In the broader context of O,O′-bidentate ligands, such structure–property relationships are essential for understanding and controlling metal–ligand interactions in coordination complexes.

#### 2.1.6. Electronic Descriptors

The electronic properties of β-diketones, generally represented as R^1^C(O)CHC(OH)R^2^ in their enol form, are strongly governed by the nature of the substituents R^1^ and R^2^. These substituents modulate the electron density within the chelate backbone through inductive and resonance effects, thereby influencing acidity, redox behaviour, and, ultimately, the donor properties of the corresponding β-diketonato ligands in metal complexes. Quantitative electronic descriptors are, therefore, essential for correlating substituent effects with structural, spectroscopic, and electrochemical properties. Substituents may exert electron-withdrawing or electron-donating effects via σ-induction (e.g., CF_3_, other perfluoroalkyl groups, or alkyl substituents) and/or π-resonance interactions (e.g., phenyl, thienyl, furyl, or alkoxy substituents) [12,13]. Electron-withdrawing groups reduce electron density on the β-diketonato backbone and enhance ligand acidity, whereas electron-donating groups increase electron density and generally decrease acidity. Because efficient electronic communication exists between both substituents and the conjugated β-diketone framework, their effects are commonly treated as additive. Consequently, descriptor values are often expressed as the sum of the individual substituent parameters, enabling straightforward comparison across ligand series [49].

The electron-donating (or electron-withdrawing) character of the β-diketone can be described by the electron-donating (or electron-withdrawing) character of the substituent groups (Hammett [52], group electronegativity [53,54,55]), or by the whole β-diketone (p*K*_a_ [12,22,28,29,30] or Lever electronic parameter [56]). The values of these electronic descriptors for different beta-diketones can be found in the supporting information of reference [57].

Two widely used empirical parameters to quantify substituent effects are the Gordy scale group electronegativities ($\chi_{R}$) and the Hammett meta substituent constants ($\sigma_{R}$). For β-diketones bearing two substituents, the cumulative electronic influence is typically expressed as ($\chi_{R^{1}}+\chi_{R^{2}}$) or ($\sigma_{R^{1}}+\sigma_{R^{2}}$). These summed values provide a convenient measure of the overall electron-withdrawing or electron-donating character imposed on the chelate ring. Increasing values of ($\chi_{R^{1}}+\chi_{R^{2}}$) or ($\sigma_{R^{1}}+\sigma_{R^{2}}$) correspond to increasingly electron-deficient ligand backbones. Such substituent constants are particularly useful in correlating structural parameters (e.g., C–O bond lengths), spectroscopic data (e.g., ν(C=O) frequencies), and electrochemical properties (e.g., reduction potentials) within homologous ligand families [47,49].

The acidity of the free β-diketone, expressed as its p*K*_a_ value, represents a direct experimental descriptor of the ligand’s electronic character. Lower p*K*_a_ values indicate greater stabilization of the conjugate base and, thus, stronger electron withdrawal by substituents. In coordinated systems, more acidic β-diketones generally yield more electron-poor β-diketonato ligands, which, in turn, decrease electron density at the metal centre. The p*K*_a_ parameter, therefore, serves as a practical bridge between substituent electronics and metal-centred properties, including redox potentials and catalytic reactivity [58].

A more global descriptor of ligand donor–acceptor properties is the Lever electronic parameter (E_L_), developed by A.B.P. Lever [56]. This parameter is derived from electrochemical data of reference transition metal complexes and provides a quantitative measure of a ligand’s net σ-donor and π-donor/acceptor ability. The E_L_ scale is additive and enables comparison of diverse ligand classes on a common electrochemical basis. For β-diketonato ligands, E_L_ reflects the cumulative influence of substituents on both σ-donation from the oxygen atoms and π-interactions within the chelate framework. As substituents become more electron-withdrawing, E_L_ values typically increase, consistent with stabilization of higher oxidation states in coordinated metal ions.

**Figure 4:**
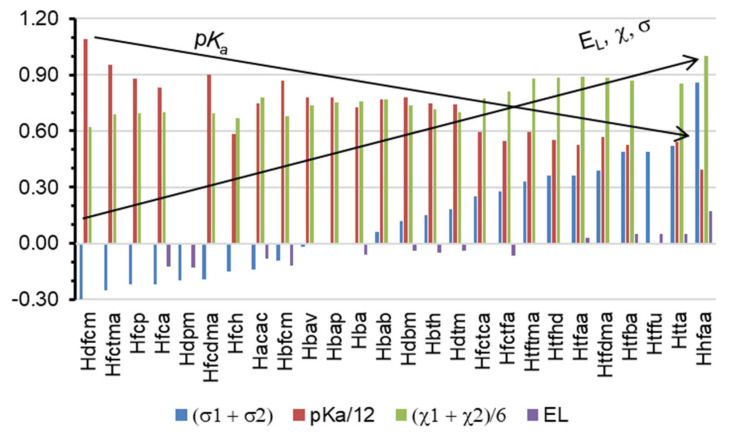
Bar graph illustrating the relationship between available substituent electronic descriptors for a series of β-diketones (R^1^COCHC(OH)R^2^). The β-diketones are shown on the *x*-axis (see List of Abbreviations for β-diketone names used) and arranged in order of increasing summed Gordy group electronegativity ($\chi_{R^{1}}+\chi_{R^{2}}$). The bars compare the corresponding summed Hammett meta substituent constants ($\sigma_{R^{1}}+\sigma_{R^{2}}$), Lever electronic parameters (E_L_), and experimental p*K*_a_ values. Increasing ($\chi_{R^{1}}+\chi_{R^{2}}$) is accompanied by parallel increases in ($\sigma_{R^{1}}+\sigma_{R^{2}}$) and E_L_, whereas the p*K*_a_ values decrease, consistent with the progressively stronger electron-withdrawing character of the substituents across the series. For clarity and direct visual comparison, the p*K*_a_ and ($\chi_{R^{1}}+\chi_{R^{2}}$) values are scaled (p*K*_a_/12 and ($\chi_{R^{1}}+\chi_{R^{2}}$)/6, respectively). The values of these electronic descriptors for different beta-diketones can be found in the supporting information of reference [57].

When β-diketones are arranged in order of increasing summed electronegativity ($\chi_{R^{1}}+\chi_{R^{2}}$), clear trends emerge; see Figure 4. The ($\sigma_{R^{1}}+\sigma_{R^{2}}$) increases in parallel with ($\chi_{R^{1}}+\chi_{R^{2}}$), the E_L_ values increase with increasing electron-withdrawing character, and the p*K*_a_ values decrease as substituents become more electron-withdrawing. These systematic relationships demonstrate that empirical substituent constants, acidity data, and electrochemical ligand parameters describe a coherent electronic framework. Together, they provide complementary tools for predicting and rationalizing trends in metal–ligand bonding strength, redox behaviour, and catalytic performance.

In the context of O,O-bidentate coordination, such descriptors are invaluable for establishing structure–property relationships and for guiding the rational design of β-diketonato ligands with tailored electronic characteristics.

#### 2.1.7. Electrochemistry

The redox behaviour of β-diketones has been investigated for several decades, providing important insight into the stability of their radical intermediates and the influence of substituents on electron transfer processes. Because β-diketones can exist in keto–enol equilibrium and possess a conjugated π-system in the enol form, their electrochemical properties are closely linked to both tautomeric structure and substituent electronics.

Early electrochemical studies demonstrated that the nature of the substituents (R^1^ and R^2^) strongly determines the reversibility of the one-electron reduction. Aromatic β-diketones, i.e., those bearing two aryl substituents, were shown to undergo a quasi-reversible one-electron reduction in aprotic solvents. The initially formed radical anion is sufficiently stabilized to allow partial re-oxidation within the time scale of cyclic voltammetry. However, it ultimately participates in follow-up chemical reactions (EC mechanism), including proton transfer and dimerization [59,60]. In contrast, purely aliphatic β-diketones, such as acetylacetone (Hacac), exhibit completely irreversible reduction waves in aprotic media. The process is kinetically controlled and influenced by a preceding chemical step, commonly associated with keto–enol interconversion [61]. Electron spin resonance spectroscopy confirmed the transient formation of unstable radical anions, consistent with their rapid chemical decay [59].

The enhanced stability of radical anions in diaryl-substituted systems is attributed primarily to resonance effects, rather than purely inductive electron withdrawal. DFT calculations support this interpretation: the LUMO of the neutral β-diketone and the spin density distribution of the radical anion show extensive π-delocalization only when two aromatic substituents are present. In such cases, the unpaired electron is distributed across R^1^–backbone–R^2^ (approximately 25:50:25), consistent with the experimentally observed reversibility (see DFT Section 2.1.5 and Figure 3) [47,49].

**Scheme 7:**
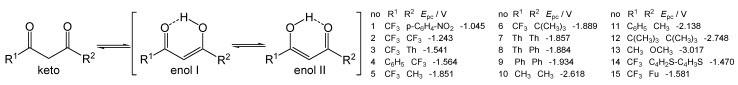
Structures of the investigated β-diketones (1–15) with their experimental cathodic peak potentials (*E*_pc_, V vs. FcH/FcH^+^ reported in [47,49]) measured in acetonitrile. Substituents span strongly electron-withdrawing to electron-donating groups, illustrating the wide modulation of reduction potentials across the series.

A systematic electrochemical study of fifteen 1,3-substituted β-diketones, R^1^COCHC(OH)R^2^, with substituents ranging from strongly electron-withdrawing (e.g., CF_3_, *p*-nitrophenyl) to electron-donating (alkyl, aryl), revealed that the reduction potentials span nearly 1.8 V in acetonitrile (referenced to FcH/FcH^+^); see Scheme 7 [47,49]. This wide potential window directly reflects differences in substituent electronic properties.

**Figure 5:**
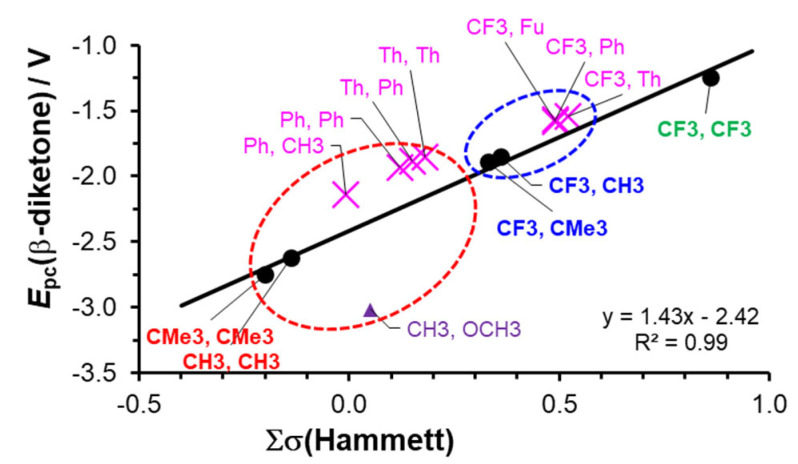
Correlation between experimental reduction potentials (*E*_pc_, V vs. FcH/FcH^+^) of β-diketones 1–15 and the summed Hammett meta substituent constants ($\sigma_{R^{1}}+\sigma_{R^{2}}$). Compounds dominated by inductive effects are shown as black circles (●), aromatic-substituted β-diketones as magenta crosses (**×**), and the ester-substituted β-diketone as a purple triangle (▲). The trend line is fitted through compounds exhibiting predominantly inductive effects. Deviations highlight resonance stabilization contributions. The cluster of data into three distinct groups, depending on the number of CF_3_ substituents, is highlighted with a red dotted circle for Group 1, no CF_3_ group; a blue dotted circle for Group 2, one CF_3_ group; and, in green, Group 3, two CF_3_ groups. Reduction data from [47,49]. Hammett constants from [52].

The reduction potentials correlate strongly with empirical electronic descriptors, such as the summed Gordy group electronegativities ($\chi_{R^{1}}+\chi_{R^{2}}$) and Hammett meta substituent constants ($\sigma_{R^{1}}+\sigma_{R^{2}}$). For β-diketones bearing only aliphatic substituents, where inductive effects dominate, excellent linear correlations (R^2^ ≈ 0.99) are observed between *E*_pc_ and these summed parameters (Figure 5). However, β-diketones containing aromatic substituents deviate systematically from the purely inductive trend. These compounds display less negative (i.e., easier) reduction potentials than predicted by inductive parameters alone. This deviation is attributed to resonance stabilization of both the neutral molecule and the radical anion. Delocalization of electron density through conjugation lowers the energy of the LUMO and increases electron affinity. The distinction between inductive and resonance effects becomes particularly evident when comparing correlations based on χ (primarily inductive) and σ (which incorporates resonance contributions). Larger deviations from linearity are observed when using χ values, confirming the important role of π-conjugation in stabilizing the reduced species [49]. An instructive example is provided by β-diketones containing ester substituents. Lone-pair donation from the ester oxygen into the conjugated system stabilizes the neutral molecule, increases electron density on the backbone, and shifts the reduction potential to more negative values than predicted by inductive considerations alone [49].

Furthermore, the data clusters into three distinct groups depending on the number of CF_3_ substituents: (Group 1) no CF_3_ group, (Group 2) one CF_3_ group, and (Group 3) two CF_3_ groups (e.g., Hfaa). This grouping highlights the exceptionally strong electron-withdrawing (inductive) character of the CF_3_ substituent; see Figure 5.

**Figure 6:**
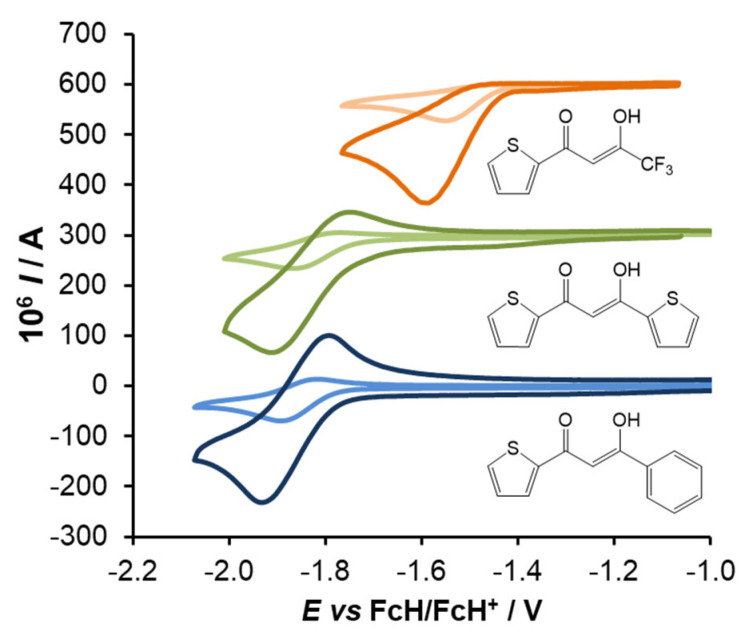
Cyclic voltammograms (vs. FcH/FcH^+^) for the indicated β-diketones at scan rates 0.20 and 2.00 V s^−1^, at 25 °C. Solvent/electrolyte = 0.1 M [^n^Bu_4_N][PF_6_]/CH_3_CN. Increased reversibility is obtained at higher scan rates only for the diaryl-substituted systems. Adapted from [49] (open access).

Cyclic voltammetry performed at varying scan rates confirms that radical anion stability depends critically on substituent conjugation. β-Diketones bearing two aromatic groups exhibit quasi-reversible behaviour at high scan rates, with peak current *i*_pa_/*i*_pc_ ratios approaching unity. In contrast, β-diketones containing one or no aromatic substituents remain electrochemically irreversible, even at fast scan rates (Figure 6).

**Figure 7:**
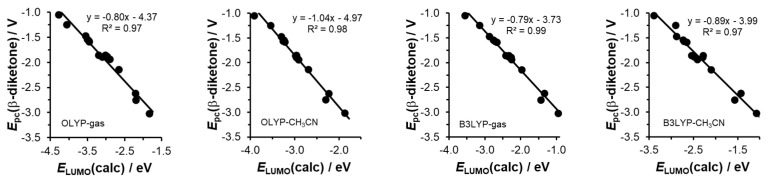
Linear correlation between experimental reduction potentials (*E*_pc_, V vs. FcH/FcH^+^) of β-diketones 1–15 (Scheme 7) and DFT-calculated LUMO energies obtained by the indicated DFT method, demonstrating frontier orbital control of the reduction process. Adapted from [49] (open access).

A strong linear relationship is observed between the experimental reduction potentials (*E*_pc_) and DFT-calculated LUMO energies for all fifteen β-diketones (R^2^ = 0.97–0.99 depending on the DFT method; Figure 7). Since reduction corresponds to population of the LUMO, this correlation provides a direct molecular orbital interpretation of the electrochemical data. Other global descriptors, such as electrophilicity index (ω) and electron affinity (EA), show similar but slightly weaker correlations. These results confirm that substituent-induced modulation of LUMO energy is the primary electronic factor governing reduction behaviour in β-diketones.

In addition to reduction, the anodic oxidation of three selected β-diketones (acetylacetone, benzoylacetone, and ethyl-4,4,4-trifluoroacetoacetate) has been investigated in aqueous ethanol at basic pH. A single irreversible anodic peak is observed, corresponding to a one-electron oxidation to a radical cation. The oxidation potential shifts to more negative values with increasing pH, consistent with proton participation in the oxidation mechanism. Under basic conditions (pH 10), oxidation is facilitated due to deprotonation equilibria. A linear correlation between the experimentally measured oxidation potentials (0.645, 0.669, and 0.795 V versus SCE as scan rate 0.400 Vs^−1^) and DFT-calculated HOMO energies (0.261, 0.265 and 0.307 eV) was demonstrated. Since oxidation involves electron removal from the HOMO, this relationship further substantiates the frontier molecular orbital control of β-diketone redox chemistry [50].

#### 2.1.8. Spectroscopic Properties

Spectroscopic techniques provide direct insight into the tautomeric equilibrium, electronic structure, and substituent effects of β-diketones. In particular, NMR and UV–visible spectroscopy are valuable for probing keto–enol equilibria and the conjugated π-system that underpins both their coordination chemistry and redox behaviour.

##### NMR Spectroscopy

^1^H NMR spectroscopy is especially informative for distinguishing between the keto and enol tautomers of β-diketones. In the keto form, the methylene protons (–C(O)–CH_2_–C(O)–) typically resonate in the region δ ≈ 3.0–5.5 ppm (depending on substituents and solvent). In contrast, the enol tautomer displays a characteristic vinylic methine proton (–C(O)–CH=C(OH)–) at lower field, generally in the range δ ≈ 5.5–7.0 ppm [12,13,41,43]. These resonances are usually well separated from substituent signals, allowing straightforward identification and quantification of tautomeric ratios.

A distinctive feature of the enol form is the presence of a strongly hydrogen-bonded OH proton engaged in intramolecular O–H···O hydrogen bonding. This signal typically appears as a broad singlet at very low field δ ≈ 14–16 ppm [41], reflecting the strength of the six-membered chelated hydrogen bond. The position and sharpness of this resonance are sensitive to solvent polarity, concentration, and temperature. For β-diketones bearing unsymmetrical substituents, multiple enol orientations are possible (Scheme 2). However, rapid interconversion between these forms leads to time-averaged signals on the NMR time scale under normal conditions. As a result, a single averaged methine resonance is usually observed [16].

Importantly, sample preparation and history can influence the observed keto–enol distribution. Freshly prepared solutions may exhibit higher apparent keto content, while aged samples approach thermodynamic equilibrium. This sensitivity has been exploited to study keto–enol interconversion kinetics by time-resolved ^1^H NMR spectroscopy [12,13,43]. Solvent effects are also significant: non-polar solvents generally favour the enol tautomer due to stabilization of the intramolecular hydrogen bond, whereas polar protic solvents may shift the equilibrium toward the keto form.

Additional structural information from ^13^C NMR includes characteristic carbonyl resonances for the keto form (typically δ ≈ 190–205 ppm) and conjugated C–O/C=C signals in the enol form. These features complement the diagnostic ^1^H NMR data discussed above. (See the Sections Structural Information from NMR Spectroscopy and Structural Information from IR Spectroscopy, for further vibrational and multinuclear NMR characteristics.)

##### UV–Visible Spectroscopy

The UV–visible spectra of β-diketones are dominated by electronic transitions associated with the conjugated π-system of the enol tautomer. For acetylacetone (2,4-pentanedione), an intense, broad absorption band is observed in the range 263–274 nm in various solvents. This transition is assigned to a π → π* excitation of the conjugated enol system and is typically structureless and of high molar absorptivity. A weaker n → π* transition appears at slightly longer wavelength (ca. 290–300 nm), arising from excitation of non-bonding electrons on oxygen [62,63].

Similar spectral features are observed for other β-diketones, such as trifluoroacetylacetone (TFAA) and hexafluoroacetylacetone (HFAA). In these compounds, the first intense absorption band (π → π*) also lies in the 260–275 nm region, with comparable absorption coefficients. The position of this band is moderately sensitive to substituent effects: electron-withdrawing groups can slightly lower the energy of the π → π* transition, reflecting stabilization of the π* orbital. In the gas phase, and in low-temperature matrices, vibrational fine structure may be resolved within this band, whereas, in solution, it generally appears broad and featureless. Higher-energy transitions are located below 200 nm and are typically not overlapped significantly with the first π → π* band [64,65,66,67].

The dominance of the enol π-system in the UV–visible spectrum further supports the conclusion—also drawn from NMR and thermodynamic data—that the enol tautomer is the predominant form in non-polar media.

The spectroscopic characteristics of β-diketones directly influence and reflect their coordination behaviour. The strong intramolecular hydrogen bond and extended conjugation of the enol form preorganize the ligand for O,O-chelation. Upon coordination and deprotonation to form the β-diketonato anion, the conjugated π-system is further delocalized over the O–C=C–C=O framework, which can lead to shifts in both NMR and UV–visible features. Together, NMR and UV–visible spectroscopy provide complementary information on tautomerism, hydrogen bonding, substituent effects, and electronic structure. These techniques, in combination with IR spectroscopy and computational studies discussed earlier, enable a comprehensive understanding of the structure–property relationships that govern the behaviour of β-diketones as O,O-bidentate ligands.

#### 2.1.9. Metal-β-Diketonato Complexes

**Scheme 8:**
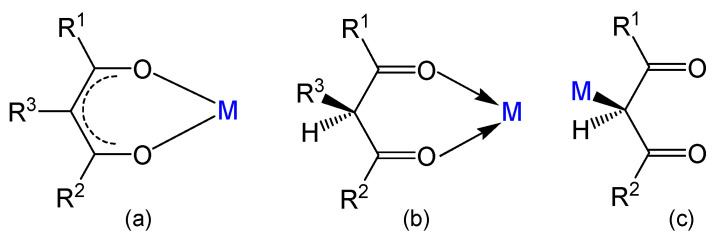
The most general binding modes in metal-β-diketonato complexes (**a**–**c**).

Excellent reviews are available on metal-β-diketonato complexes [1,6,7,8,68,69,70,71,72,73,74], indicating at least 22 different modes of coordination in mono-, di-, and polynuclear β-diketonate complexes [75]. Many complexes with alkali and alkaline earth metals, first–third row transition metals, and rare earth metals are known [8]. O,O-chelation, where the metal forms a six-membered ring with the β-diketonato ligand, is most common (Scheme 8a). Many M(β-diketonato)_n_ complexes with n = 1 (e.g., Li, Na, K, Cs), 2 (e.g., Be, Ca, Zn, Ni), 3 (e.g., Sc, V, Cr, Mn, Fe), and up to 4 (e.g., Zr, Hf, Ce), are known. Apart from the O,O-chelation form, two common forms are the keto (b) and carbon-bonded (c) species (Scheme 8) [76].

#### 2.1.10. Application

The ability of β-diketones to form stable chelate complexes through the monoanionic β-diketonato fragment underpins their broad applicability across coordination chemistry, materials science, catalysis, and medicinal chemistry. Their O,O-bidentate coordination mode, combined with tuneable electronic properties via substituent modification, makes them one of the most extensively used ligand classes for both d- and f-block elements [77,78].

##### Metal Complexation and Extraction

Deprotonation of the enol form yields the β-diketonato anion, which readily coordinates to a wide range of metal ions. This strong complexing ability forms the basis for their long-standing use in solvent extraction processes, particularly for the removal or separation of metal ions from aqueous media [28,79]. Fluorinated β-diketones, in particular, display enhanced extraction efficiency due to their increased acidity and lipophilicity [35,80,81]. In addition, many volatile metal β-diketonate complexes are employed as precursors in chemical vapour deposition (CVD) and related thin-film fabrication techniques [82,83]. Their thermal stability and volatility can be systematically tuned through ligand substitution.

##### Catalysis and Polymer Chemistry

Transition metal β-diketonate complexes are widely used as homogeneous and heterogeneous catalysts. They have been applied in oligomerization, olefin oxidation, epoxidation, and related transformations [76]. In polymer chemistry, such complexes serve as polymerization catalysts, or as additives that modify polymer properties, including UV stability and oxidative resistance [84]. Beyond catalysis, β-diketones themselves are also valuable synthetic intermediates in organic chemistry, particularly in the construction of heterocycles such as pyrazoles, isoxazoles, and triazoles [85,86]. Their 1,3-dicarbonyl framework is a versatile building block for numerous functionalized scaffolds [87,88].

##### Luminescent and Functional Materials

β-Diketones play a central role in the design of luminescent coordination compounds. Lanthanide β-diketonate complexes have been extensively studied for applications in lighting, laser materials, and photonic technologies [89,90,91,92]. Incorporation of perfluorinated substituents significantly enhances luminescence efficiency by suppressing non-radiative multiphonon relaxation processes [93].

Difluoroboron bis-β-diketonate complexes constitute another important class of photofunctional materials. These compounds are characterized by high molar absorption coefficients, strong fluorescence emission, and, in some cases, two-photon absorption properties [94]. As a result, they have been investigated for use in light-emitting diodes (OLEDs) [95,96], semiconducting materials, photochemical and -chromic applications, conjugated polymers, near-IR probes, and oxygen and mechanical sensors [97].

##### Biological and Pharmaceutical Relevance

The β-dicarbonyl motif is present in numerous biologically active molecules and natural products. Naturally occurring 1,3-diketones have been isolated from sources such as vanilla beans, eucalyptus leaves, sunflower pollen, and liquorice roots. Many exhibit notable biological activities, including antimicrobial, antiviral, antifungal, and antitumour properties [41,98,99].

Dibenzoylmethane (DBM), for example, is a naturally occurring β-diketone recognized for antioxidant and anticancer activity, demonstrating inhibition of tumour growth and metastasis in various in vitro and in vivo models [100]. More broadly, the β-diketone scaffold forms the structural basis of several pharmacologically relevant compound classes. Curcuminoids, which also contain the β-diketone motif, exhibit anticancer, antibacterial, anti-inflammatory, and neuroprotective properties [101].

In medicinal chemistry, 1,3-diketones serve as versatile intermediates for the synthesis of heterocyclic pharmacophores and have been associated with analgesic, antimicrobial, antidiabetic, anti-inflammatory, and antitumour activities [35,102].

##### Analytical and Industrial Uses

Beyond catalysis and medicine, β-diketones find application in analytical chemistry due to their selective complexation properties, enabling metal preconcentration and detection [28]. They have also been employed as fuel additives [103], lubricant components, UV-protective additives in cosmetic formulations [104], and functional modifiers in polymer materials.

Owing to their strong chelating ability, adjustable electronic properties, rich tautomeric chemistry, and compatibility with a wide range of metal ions, β-diketones remain among the most versatile and widely used O,O-bidentate ligands. Their impact spans coordination chemistry, materials science, catalysis, analytical chemistry, and drug discovery. The breadth of applications outlined above underscores why β-diketones continue to attract sustained interest, more than a century after their introduction into coordination chemistry.

### 2.2. Imino-β-Diketones and Di-Imino-β-Diketones (N,O- and N,N′-Bidentate Ligands)

Replacement of one or both carbonyl oxygen atoms in β-diketones by imine nitrogen donors produces structurally related ligand classes with distinct donor properties and electronic characteristics (Scheme 1). Mono-imine derivatives, commonly referred to as imino-β-diketones or enaminones, typically act as N,O-bidentate ligands, whereas the corresponding di-imine analogues (β-diketiminates or 1,3-diketimines) behave as N,N′-chelating ligands. Owing to their conjugated backbones and readily tuneable substituents, these systems represent an important link between classical β-diketonate ligands and Schiff base coordination frameworks.

**Scheme 9:**
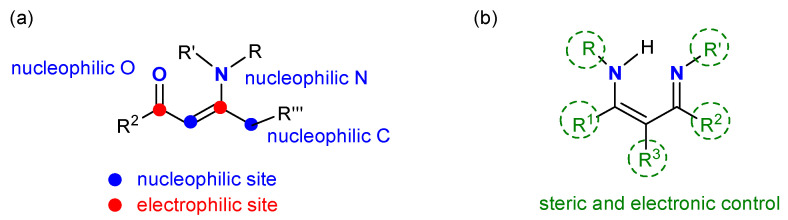
(**a**) Structure and reactive sites of enaminone, (**b**) structure and modification of substituent groups for steric and electronic control of β-diketiminates.

The conjugated N–C=C–C=O motif of enaminones gives rise to a polarized π-system that contains several chemically distinct reactive centres. In general, the carbonyl carbon (C-1) and the β-carbon (C-3) display electrophilic character, while nucleophilic sites may include the carbonyl oxygen, the enamine nitrogen, and the α-carbon (C-2) within the conjugated chain (Scheme 9). This dual electrophilic–nucleophilic behaviour originates from resonance delocalization across the enaminone framework, which combines the electronic features of both enamines (nucleophilic) and α,β-unsaturated carbonyl compounds (electrophilic). Consequently, enaminones are versatile synthetic intermediates, capable of reacting with a wide range of nucleophiles and electrophiles to generate diverse acyclic, carbocyclic, and heterocyclic structures, as discussed in references [2,105].

In contrast to enaminones, β-diketiminates (1,3-diketimines, often referred to as “NacNac” ligands) represent fully nitrogen-substituted analogues of β-diketones and constitute a highly versatile class of N,N′-bidentate ligands. Their general framework, RN(H)–C(R^1^)=C(R^3^)–C(R^2^)=NR′, allows extensive steric and electronic tuning through variation of both the imine substituents (R, R′) and the ligand backbone (R^1^–R^3^). When coordinated to a metal, substitution at the nitrogen atoms primarily controls steric encumbrance around the metal centre, while modification of the backbone substituents exerts a stronger influence on the electronic properties of the ligand (Scheme 9). In particular, electron-withdrawing groups can significantly alter metal-centred redox properties and reduce metal–ligand back-donation, whereas bulky substituents promote the formation of low-coordinate, mononuclear complexes by restricting the N–M–N bite angle [3].

A notable feature of β-diketiminates is their ability to stabilize metal centres across a wide range of oxidation states, including low-valent and highly reactive species, through a combination of strong σ-donation and delocalized π-interactions. Consequently, β-diketiminato complexes are known for a broad range of s-, p-, d-, and f-block elements and support diverse coordination environments and reactivity patterns [5]. This versatility has led to their widespread use as supporting ligands in coordination chemistry, particularly in systems requiring stabilization of reactive intermediates or unusual bonding motifs. The structural and electronic flexibility of these ligands, thus, complements that of β-diketones and enaminones, making them well suited for comparative analysis within a unified 1,3-dicarbonyl-derived ligand framework.

#### 2.2.1. Structure and Tautomerism

##### Enaminones (N,O Donors)

Imino-β-diketones, commonly referred to as enaminones or β-amino-α,β-unsaturated ketones, may be described as vinylogous amides featuring a conjugated N–C=C–C=O fragment. In this review, the focus is placed on systems of general form R^1^-C(NR′R)=CH-C(O)-R^2^ with R′ = H, which are structurally derived from β-diketones by condensation with amines.

**Scheme 10:**
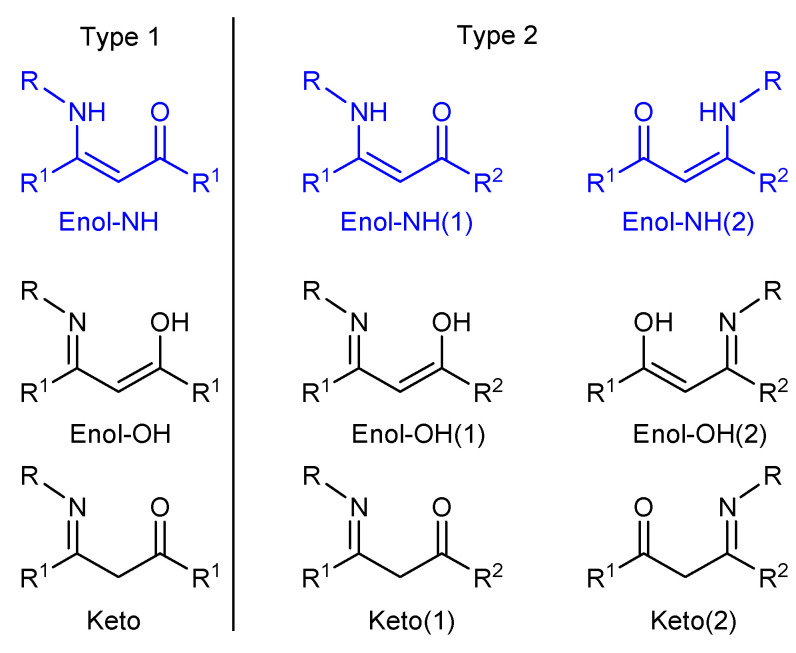
Possible keto and enol tautomers of amino-substituted β-amino-α,β-unsaturated ketones R^1^C(O)CH=C(NHR)R^2^, illustrating geometric and prototropic variations. The enol–NH (top, shown in blue) is the most general form.

Like β-diketones, enaminones exhibit tautomerism. Three principal forms can be considered, the keto form (carbonyl retained), the enol form, and the enol–amine (enol–NH) form; see Scheme 10. Although multiple geometrical isomers are theoretically possible (particularly when R^1^ ≠ R^2^), experimental evidence shows a strong preference for the enol–NH tautomer in both solution and the solid state [106,107,108].

The predominance of the enol–NH form is attributed to intramolecular N–H···O hydrogen bonding and conjugation across the C=C–C=O framework. When aromatic substituents are present, additional π-delocalization further stabilizes this arrangement.

##### β-Diketiminates (N,N′ Donors)

**Scheme 11:**
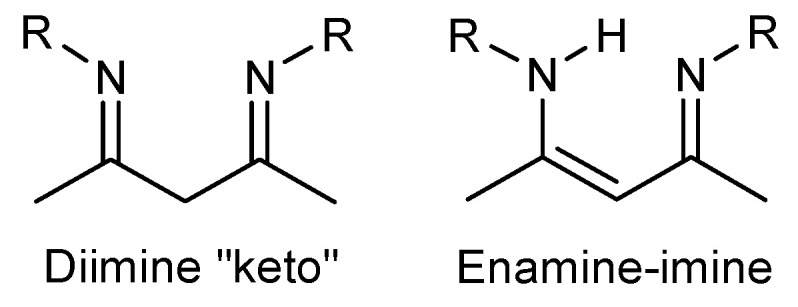
Tautomeric forms of 1,3-diketimines (β-diketiminates): diimine versus enamine–imine configurations.

Di-imino derivatives (1,3-diketimines), often termed β-diketiminates or “HNacNac” ligands, are formally obtained by replacing both carbonyl oxygen atoms of a β-diketone with NR groups. The parent framework exists in equilibrium between two forms, the diimine (keto-like) form, RN=C(Me)–CH_2_–C(Me)=NR, and the enamine–imine form, RN(H)–C(Me)=CH–C(Me)=NR; see Scheme 11. The enamine–imine tautomer is generally favoured due to extended conjugation and stabilization by intramolecular hydrogen bonding [109].

#### 2.2.2. Synthesis

As this review focuses primarily on the structural and physicochemical properties of O,O′-, N,O-, and N,N′-Bidentate ligands, with the focus on β-enaminones and β-diketiminates in this section, their synthesis will be addressed only briefly. Comprehensive discussions of synthetic methodologies are available in several dedicated reviews of β enaminones [2,5,105,110] and β-diketiminates [3,4,5,111].

##### Enaminones

**Scheme 12:**
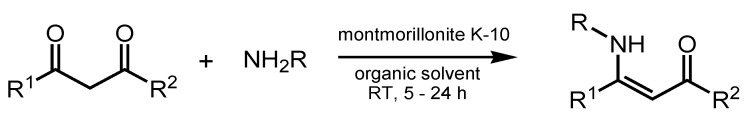
General synthetic route to amino-substituted β-amino-α,β-unsaturated ketones (enaminones) via condensation of a 1,3-dicarbonyl compound with a primary amine under catalytic or solvent-assisted conditions, as described in reference [116].

Enaminones are typically prepared via condensation of β-diketones or β-ketoesters with primary or secondary amines [112]. The reaction may proceed under reflux without catalyst [108], in the presence of Brønsted or Lewis acid catalysts [113], or on solid supports such as silica [114], alumina, or montmorillonite K-10 [115,116] (Scheme 12). Solid-supported methods have proven particularly efficient, affording high yields (up to ~93%) under mild conditions and often at room temperature [108,116]. These protocols minimize energy input and simplify purification, which is typically achieved by recrystallization or sublimation.

##### β-Diketiminates

**Scheme 13:**
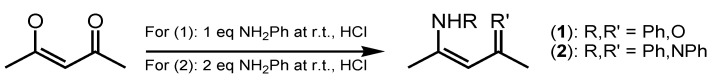
Stepwise condensation of acetylacetone with aniline to form (1) the mono-imine enaminone and (2) the di-imine β-diketiminate ligand under acidic conditions, as described in reference [117].

β-Diketiminates are synthesized analogously to enaminones by double condensation of a β-diketone (e.g., acetylacetone) with two equivalents of a primary amine under acidic conditions to promote dehydration. Mono-condensation affords the enaminone, whereas use of two equivalents of amine yields the 1,3-diketimine [117] (Scheme 13).

#### 2.2.3. Structural Characterization

##### NMR Spectroscopy

^1^H NMR spectroscopy provides clear evidence for tautomeric preferences. The vinylic (=CH–) proton of enaminones typically resonates between 4.8 and 6.2 ppm, depending on substitution. Phenyl substitution shifts this resonance downfield, consistent with enhanced conjugation. A broad resonance at 10–13 ppm is characteristic of the N–H proton engaged in intramolecular hydrogen bonding, supporting assignment of the enol–NH tautomer. Integration confirms the presence of a single methine proton, excluding keto forms in solution. ^13^C NMR spectra display signals consistent with retained carbonyl (C=O) functionality (~190–200 ppm) and amine (C-N) carbons (~155–165 ppm), further corroborating the enol–NH structure [116] (Scheme 10).

##### Infrared Spectroscopy

IR spectra exhibit strong N–H stretching bands in the 3000–3300 cm^−1^ region, consistent with hydrogen-bonded amine functionality. The presence of a carbonyl stretching vibration confirms that enol–NH, rather than enol–OH, or fully keto structures, predominates [116].

##### Solid-State Structures

Enaminones (N,O donors)

**Figure 8:**
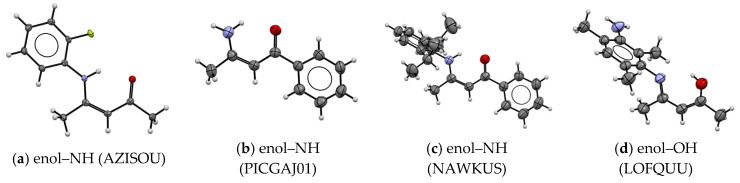
Representative examples of published enaminone structures (CSD [42] reference code below each structure).

Available crystallographic data reveal the enol–NH configuration, stabilized by the intramolecular NH...O_CO_ hydrogen bond, for normal enaminones. Only one enol–OH structure (LOFQUU) could be found; see Figure 8. Contrary to what was found for β-diketones, no purely keto structures have been reported for these systems [42]. In phenyl-substituted derivatives, the aryl group is oriented syn to the carbonyl group, consistent with conjugative stabilization (for example, see CSD reference codes: PICGAJ, PICGAJ01, PICGAJ02, BIVQON, BIVQUT, BIVRAA, BIVRAA01, BIVREE, HUDVAE, KUXJUI, KUXJUI01, KUXJUI02, KUXJUI03, NAWKUS, PUKQUI, RALROM, RAWPOW, RAWQEN, VAZPOC, WUHNIX, ZIJNOX, ZIJNOX01); see Figure 8b,c.

β-Diketiminates (N,N′ donors)

**Scheme 14:**
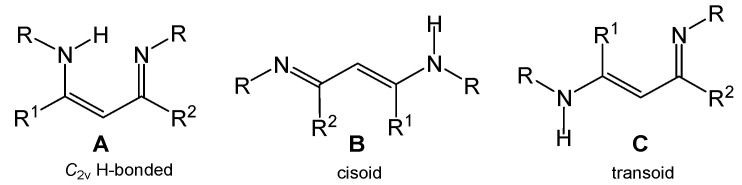
Conformers of di-imino-β-diketones (β-diketiminates).

A large number of β-diketiminate structures have been reported in the literature. As a representative example, the framework RN(H)–C(Me)=C(H)–C(Me)=NR′ is considered. This ligand can adopt several conformations (Scheme 14), including a *C*_2v_-symmetric intramolecularly hydrogen-bonded chelating form (A), as well as cisoid (B) and transoid (C) arrangements, defined by the relative orientations of the two backbone R^1^ and R^2^ substituents.

**Figure 9:**
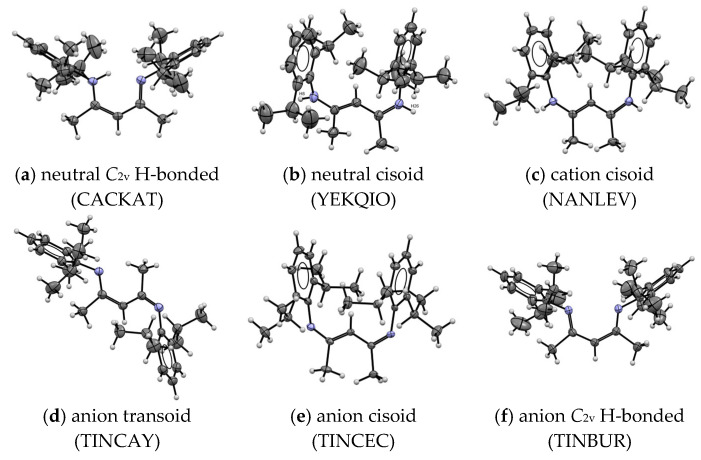
Example of different published β-diketiminate DippN(H)–C(Me)=C(H)–C(Me)=NDipp structures (CSD [42] reference code below each structure).

Analysis of crystallographic data shows that the neutral species overwhelmingly adopts the *C*_2v_-symmetric hydrogen-bonded form (A), with more than 70 reported structures exhibiting this arrangement; see Figure 9a. Only a single example (YEKQIO), in which the N–H proton (H8 and H26) is disordered over both nitrogen atoms, displays a cisoid (E) conformation with the backbone methyl groups in a cis orientation; see Figure 9b. In contrast, all structurally characterized protonated (cationic) derivatives (e.g., JOBROI, MODQUT, MODRII, NANLEV, VEBHER, YEYNAU) adopt the cisoid (B) form; see Figure 9c.

More recently, the first crystallographic characterization of metal-free β-diketiminate anions (BDI^−^) was reported (TINBUR, TINCAY, TINCEC, TINCIG) [118]. These studies on Dipp-substituted systems, [DippN(H)–C(Me)=C(H)–C(Me)=NDipp]^−^ (Dipp = 2,6-iPr_2_C_6_H_3_), in the presence of various counterions (e.g., Cs^+^, 18-crown-6, and [2.2.2]-cryptand), revealed multiple solid-state rotamers. The observed conformations include an unbound *C*_2v_-symmetric form (A), as well as cisoid (B) and transoid (C) structures (Figure 9d–f, Scheme 14).

Solution NMR studies indicate rapid interconversion between the cisoid and transoid forms, consistent with the equilibrium [(donor)Cs]^+^[transoid-BDI]^−^ ⇄ [(donor)Cs]^+^[cisoid-BDI]^−^. DFT calculations further support the accessibility of these rotamers, with relative energies within 5.2 kcal·mol^−1^ and the transoid (C) conformation identified as the lowest-energy structure. Representative structures of the neutral, cationic, and anionic DippBDI species are shown in Figure 1.

#### 2.2.4. DFT Studies and Frontier Orbitals

Density functional theory (B3LYP/6-311G(d,p)) calculations reproduce experimental geometries with good agreement and confirm that the enol–NH tautomer is energetically preferred. For phenyl-substituted systems, the lowest-energy conformer corresponds to the configuration observed crystallographically [116].

**Figure 10:**
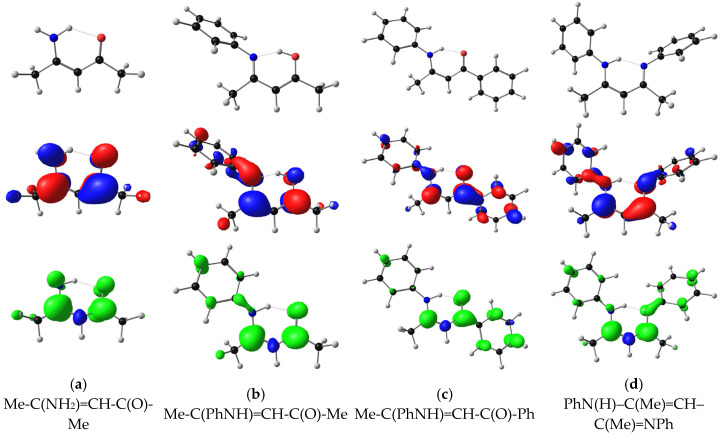
DFT-calculated optimized geometry (top), and LUMOs (middle) of representative neutral (**a**–**c**) enol–NH enaminones and (**d**) β-diketiminate, including the corresponding spin density plots (bottom) of their radical anions, illustrating delocalization across the conjugated backbone and aryl substituents. Contours of 0.05 and 0.005 e/Å^3^ were used for the MO and spin plots, respectively. Colour code of the atoms (online version): N (blue), C (grey), O (red) and H (white). Figures reproduced from DFT data reported in [116,117].

For both enaminones and β-diketiminates, the calculated LUMO of the neutral ligand is largely delocalized over the conjugated N–C=C–C=O (or N–C=C–C=N) backbone and extends into attached phenyl rings when present [116,117]; see Figure 10.

Spin density plots of the radical anions show that the added electron is distributed across the conjugated framework, particularly in aryl-substituted systems, highlighting efficient electronic communication between substituents and the chelate backbone [116,117]; see Figure 10.

#### 2.2.5. Electrochemistry

**Figure 11:**
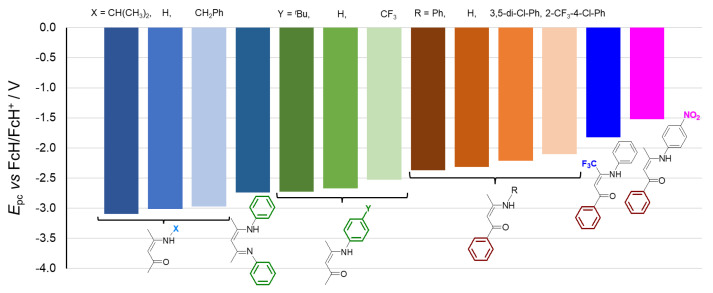
Influence of phenyl substitution and para-substituent variation on the reduction potentials *E*_pc_ of enaminones (N,O) and β-diketiminate (N,N’), highlighting systematic electronic effects. Data from [116] and [117].

Similar to β-diketones, cyclic voltammetry reveals a single chemically irreversible reduction process for most enaminones. However, phenyl-containing derivatives generally exhibit partial electrochemical reversibility at higher scan rates, indicating transient stabilization of the radical anion. Substituent effects follow a systematic trend: increasing electron-withdrawing character shifts reduction potentials to less negative values (facilitating reduction). For example, for the enaminone series CH_3_–C(O)–CH=C(HN-*p*-Y-C_4_H_4_)–CH_3_, the reduction potential linearly relates to the *para*-Hammett constant, σ_p_, with R^2^ = 1.00 where Y (reduction potential *E*_pc_ in V vs. FcH/FcH^+^, σ_p_) = *^t^*Bu (−2.726, −0.2), H (−2.673, 0), CF_3_ (−2.526, 0.57). Comparing *E*_pc_ of Ph–C(O)–CH=C(HNPh)–**CH_3_** (−2.370) and Ph–C(O)–CH=C(HNPh)–**CF_3_** (−1.826), the electro- withdrawing power of the CF_3_ compared to CH_3_ group clearly shifts more positive. Phenyl substitution (electronic inductive and resonance effect) and para-substituent variation (electronic inductive effect) clearly modulate redox behaviour [116]; see Figure 11.

The influence of the electronegativity (EN) of O in the carbonyl group versus N in the imine group on the reduction potential of the ligand can also be explained in terms of the difference in the EN of O (EN = 3.44) and N (EN = 3.04), then comparing related O,O′-, N,O-, and N,N′-bidentate ligands. Examples of the influence of imine substitution at one carbonyl in a β-diketones to obtain imino-β-diketones include the following: Comparing *E*_pc_ of Hacac, CH_3_–C(O)–CH=C(**OH**)–CH_3_ (−2.618 [49]) and (CH_3_–C(O)–CH=C(**NH_2_**)–CH_3_) (−3.017 [116]), it is clear that the more electron-donating power (less electronegative) of N compared to O causes the N-containing ligand to be reduced at a lower, more negative potential; similarly, imino substitution in Hba, Ph–C(O)–CH=C(**OH**)–CH_3_ (−2.138 [49]) and (Ph–C(O)–CH=C(**NH_2_**)–CH_3_) (−2.317 [116]) shows the same trend. Secondly, the lower *E*_pc_ of the di-imino-β-diketone PhN(H)–C(Me)=CH–C(Me)=**CO** (−2.617 V [116]) compared to *E*_pc_ of the mono-imino-β-diketone PhN(H)–C(Me)=CH–C(Me)=**NPh** (−2.741 V [117]) illustrates the progressive shift to more negative potentials due to the higher electron-donating power of N compared to O.

**Figure 12:**
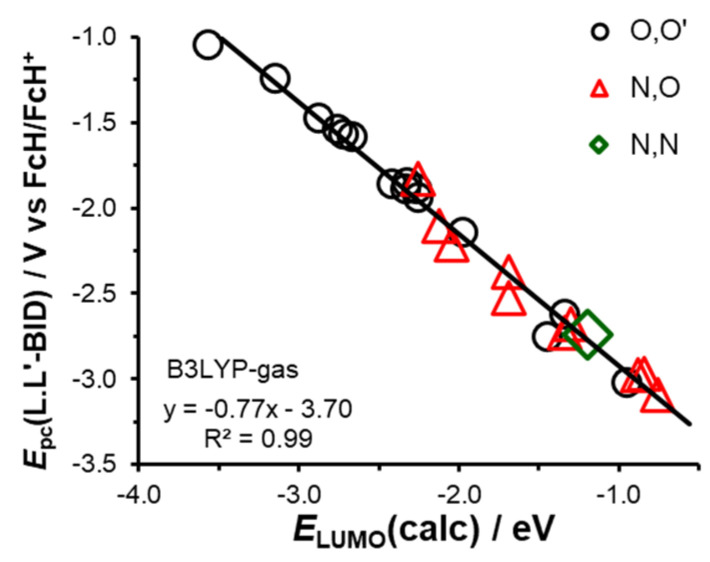
Correlation between experimental reduction potentials (V vs. FcH/FcH^+^) and DFT-calculated LUMO energies for O,O′-, N,O-, and N,N′-bidentate ligands, demonstrating a unified frontier orbital-controlled redox trend. Reproduced using data from [117] (open access).

A strong linear relationship for enaminones is observed between experimental reduction potentials (*E*_pc_) and calculated electronic descriptors, including LUMO energy (R^2^ ≈ 0.95), electron affinity (R^2^ ≈ 0.92), electrophilicity index ω (R^2^ ≈ 0.94), and Mulliken electronegativity χ (R^2^ ≈ 0.89) [116]. Remarkably, β-diketones (O,O′), enaminones (N,O), and β-diketiminates (N,N′) fall on a common linear correlation between reduction potential and calculated LUMO energy (R^2^ ≈ 0.99), demonstrating that the frontier orbital energy, as influenced by the electronic effect of different substituents, primarily governs ligand-centred reduction across donor sets [117]; see Figure 12.

**Scheme 15:**
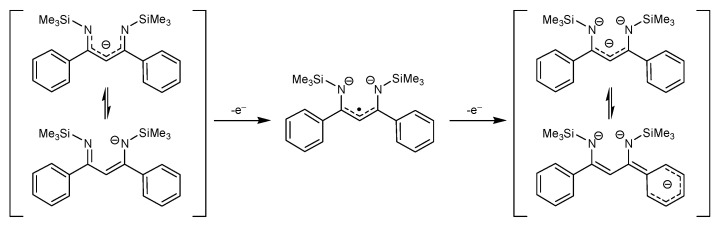
Successive 1 e^−^ reductions of a coordinated N,N′-bis(trimethylsilyl)-β-diketiminate anion [120].

While the free di-imino-β-diketone showed one irreversible reduction in the solvent window of the solvent used in the electrochemical experiment [117], it was shown that a coordinated di-imino-β-diketone anion can further be reduced in two successive one-electron steps (Scheme 15). This redox-active (non-innocent) behaviour of β-diketiminates (L) was first demonstrated by Lappert and co-workers. In the vast majority of metal β-diketiminate complexes, the ligand is present in its monoanionic form (L^−^) [5]. However, studies by the Lappert group revealed that, in certain ytterbium cluster compounds, β-diketiminato ligands can adopt multiple oxidation states, including L^−^, L^2−^, and L^3−^. For example, the cluster [Yb{(μ-L)Li(THF)}_2_], where L = (N(SiMe_3_)C(R))_2_CH (R = Ph or C_6_H_4_Ph-4), contains singly reduced dianionic ligands [119]. In a related trinuclear complex, [(YbL)_3_(THF)] (L = (N(SiMe_3_)C(Ph))_2_CH), both L^−^ and L^3−^ ligand oxidation states are present alongside Yb(II) and Yb(III) centres [120]. X-ray crystallographic analysis, supported by DFT calculations, indicates that reduction of the monoanionic ligand involves electron delocalization across the conjugated backbone, with partial localization on the phenyl substituents (Scheme 1). The stepwise one-electron reduction of the coordinated monoanionic β-diketiminate [(N(SiMe_3_)C(Ph))_2_CH]^−^, leading to these unusual ligand oxidation states, is illustrated in Scheme 1 [120].

#### 2.2.6. Metal-Imino- and Metal Di-Imino-β-Diketonato Complexes

##### Metal Enaminones

Metal complexes of imino-β-diketonates (enaminones) are widely reported, with the ligand most commonly coordinating in a chelating κ^2^-(N,O) mode to form a six-membered metallacycle. This binding mode allows additional coordination by ancillary ligands, resulting in a broad range of coordination numbers and geometries. Reported coordination numbers span from three to eight, depending on the metal centre, ligand substituents, and the presence of bridging groups.

Lower coordination numbers (3–4) are typical for p-block and late transition metals (e.g., Sn, Ge, Cu, Pd, Ni, Pt, Zn, Al, Mg), whereas higher coordination numbers (5–7) are frequently observed for early transition metals and lanthanides (e.g., V, Cr, Y, Sm, Nd, La), often involving bridging ligands or multinuclear assemblies. Very high coordination numbers (7–8) are largely restricted to f-block elements (e.g., Yb, Tb, Lu, Th, U, Pu), reflecting their larger ionic radii.

Homoleptic complexes of the type M(II)(enaminone)_2_ are common (e.g., M = Sn, Cu, Cr, Pd, Fe, Ni, Co, Pt, Zn), while M(III)(enaminone)_3_ species are less frequently encountered but well documented for metals such as Al, V, Cr, Fe, Ru, Zr, Y, Yb, and Bi. Complexes with higher ligand coordination, such as M(IV)(enaminone)_4_, are rare and typically limited to large metal centres (e.g., Th, U, Pu) capable of accommodating multiple chelating ligands [42]. No structurally characterized M(V)(enaminone)_5_ complexes have been reported in the Cambridge Structural Database (CSD [42]), likely due to steric and electronic constraints.

##### Metal β-Imino Complexes

**Scheme 16:**
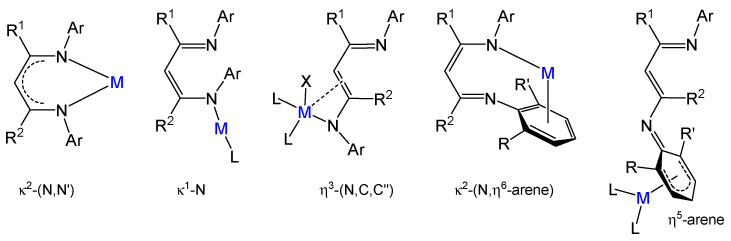
Known coordination modes of metal β-imino complexes [118].

Comprehensive reviews on metal β-diketiminate complexes are available in the literature [3,4], highlighting the diverse coordination modes accessible to these ligands [118] (Scheme 16). β-Diketiminates (di-imino-β-diketones) predominantly coordinate in a chelating κ^2^-(N,N′) mode, generating a six-membered metallacycle analogous to β-diketonates. Their strong σ-donor character, combined with steric tuneability, enables stabilization of a wide range of metals across the periodic table, including Group 1 (Li, K), Group 2 (Be, Mg, Ca, Sr), Group 13 (B, Al, Ga, In, Tl), Group 14 (Si, Ge, Sn), and transition metals from Groups 5–12 (e.g., V, Cr, Mn, Fe, Co, Ni, Cu, Zn, Rh, Ir, Ag), as well as both low- and high-valent oxidation states [4].

Structurally, M(II)(β-diketiminate)_2_ complexes are well represented. Selected examples include Cu (CSD reference EKABAU, VEPZEX–VEQBAW, XOVNEC), Ni (GEKZED, VEQVUI), Co (REKVUA, REKWAH), Zn (QABYAV), and Pd (VACHIU), featuring both NH-substituted and aryl-substituted ligands. In addition, more than 60 structures of N-aryl-substituted β-diketiminate complexes have been reported for metals such as Cr, Zn, Yb, Mn, Mg, Fe, Cu, Sr, Ba, Al, Ca, Ni, and Y, highlighting the widespread applicability of these ligands [42].

Higher oxidation state complexes of the type M(III)(β-diketiminate)_3_ are less common, but span a diverse range of metals, including transition metals and lanthanides. Representative examples include U (FUDPIG), Cr (IJEVUP, IJEWAW), Fe (NEMXEJ, NEMXIN), Al (QEYXIB), and several lanthanides, such as Yb (QUSGER, QUSGIV, QUSGOB), Nd (TUNLOE, TUNLEU, TUNLUK), Sm (TUNLIY, WEWNIV), Gd (WEWNOB), and Lu (XUBFAE) [42].

Complexes with four β-diketiminate ligands, M(IV)(β-diketiminate)_4_, are rare and typically require small N-substituents to reduce steric congestion, for example, a Zr complex with NH-substituted ligands (BEDMIH). The absence of M(V)(β-diketiminate)_5_ species in the CSD [42] reflects steric limitations, as well as the electronic constraints associated with accommodating five bulky chelating ligands around a single metal centre.

In addition to mononuclear species, β-diketiminates frequently support low-coordinate, univalent, and multinuclear complexes, owing to their steric bulk and electronic flexibility. Bridging coordination modes (μ) and the stabilization of reactive intermediates are also commonly observed, particularly for early transition metals and main-group elements.

#### 2.2.7. Selected Applications

##### Coordination Chemistry

Enaminones function as N,O-bidentate ligands analogous to β-diketonates. They readily coordinate to transition metals such as Ru(III) [121], Cu(II) [108], Ni(II), and Pt(II), forming stable chelate complexes [122]. Compared to β-diketonates, β-ketoiminates often exhibit enhanced thermal stability and reduced substrate oxidation during film deposition processes.

β-Diketiminates (N,N′ donors) are widely used in organometallic chemistry as sterically and electronically tuneable supporting ligands, particularly for early transition metals and main-group elements [3]. Their strong chelation and delocalized backbone provide kinetic stabilization of reactive metal centres [3]. β-Diketiminato metal complexes have found widespread application in catalysis, where both high-valent and low-valent species exhibit significant activity. Representative systems based on main-group (e.g., Mg, Ca, Al), transition metal (e.g., Sc, Ti, Cr, Fe, Zn), and lanthanide centres have been applied in processes such as hydroamination, olefin polymerization, and copolymerization of CO_2_ with epoxides [4].

##### Chemical Vapour Deposition (CVD)

Metal β-ketoiminate complexes have been explored as precursors for CVD processes [106]. Fluorinated derivatives, in particular, improve volatility and thermal robustness, making them attractive for thin-film fabrication.

##### Organic and Medicinal Chemistry

Enaminones serve as versatile intermediates in alkylation, acylation, and Grignard-type transformations. Biologically, they have been investigated for anticonvulsant activity, with structure–activity relationships influenced by lipophilicity, steric factors, and hydrogen bonding capability [105].

Imino-β-diketones and β-diketiminates represent nitrogen-containing analogues of classical β-diketonate ligands. While maintaining the conjugated chelate backbone characteristic of O,O′ systems, substitution of oxygen by nitrogen modifies donor strength, redox behaviour, hydrogen bonding patterns, and coordination versatility. Their structural adaptability, predictable electronic trends, and compatibility with computational descriptors make them valuable platforms for studying structure–property relationships across O,O′-, N,O-, and N,N′-bidentate ligand classes.

## 3. Conclusions

This review examined the structural and electronic properties of three closely related classes of chelating ligands: β-diketones (O,O′ donors), imino-β-diketones (enaminones, N,O donors), and di-imino-β-diketones (β-diketiminates, N,N′ donors). Together, these ligand systems illustrate how systematic replacement of oxygen by nitrogen within a conjugated 1,3-dicarbonyl framework modulates coordination behaviour, redox properties, and overall electronic structure while preserving the fundamental chelate motif.

β-Diketones represent the prototypical O,O′-bidentate system, characterized by keto–enol tautomerism and strong preference for the enolate form upon coordination. Their ability to delocalize negative charge over the O–C=C–C=O backbone underpins their stability, predictable coordination modes, and widespread use in coordination chemistry, catalysis, and materials science. Substituent effects on the β-diketone scaffold enable fine control over acidity, volatility, and metal–ligand bond strength, which has been exploited extensively in metal extraction, homogeneous catalysis, and chemical vapour deposition (CVD) processes.

Replacement of one carbonyl oxygen with an imine nitrogen affords imino-β-diketones (enaminones), which retain the conjugated backbone but introduce asymmetry in donor strength and hydrogen bonding capability. These N,O-bidentate ligands predominantly adopt the enol–NH tautomer in both solution and solid state, stabilized by intramolecular hydrogen bonding and π-delocalization. Spectroscopic, crystallographic, and DFT investigations consistently demonstrate that their frontier molecular orbitals are delocalized across the entire conjugated framework and extend into aryl substituents when present. Electrochemical studies reveal that their reduction potentials correlate strongly with computed LUMO energies and computed global and local reactivity descriptors, underscoring the predictive value of computational approaches for ligand design.

Further substitution of both carbonyl groups yields β-diketiminates (1,3-diketimines), N,N′-chelating ligands often referred to as “HNacNac” systems. These ligands typically favour the enamine–imine tautomer and exhibit highly delocalized π-systems comparable to those of β-diketonates, yet with enhanced σ-donor character from nitrogen. As a result, β-diketiminates have become prominent supporting ligands in organometallic and main-group chemistry, where their steric and electronic tuneability stabilizes reactive metal centres. Importantly, comparative electrochemical analysis shows that O,O′-, N,O-, and N,N′-bidentate systems follow a common linear relationship between reduction potential and calculated LUMO energy, indicating that the redox behaviour across these ligand families is governed primarily by the energy and distribution of the lowest unoccupied molecular orbital, rather than by donor set alone.

A key outcome of this review is the identification of clear and transferable structure–property relationships across the β-diketone-derived ligand families. Direct comparison of O,O-, N,O-, and N,N′-donor systems demonstrates how progressive donor substitution systematically modifies electronic structure, steric demand, and coordination behaviour. In particular, while β-diketonates readily form highly coordinated complexes such as M(IV)(β-diketonato)_4_ (e.g., Zr, Hf, Ce), the corresponding enaminone and β-diketiminate analogues show a marked reduction in such species. M(IV)(enaminone)_4_ complexes are rare and largely restricted to large f-block centres, whereas M(IV)(β-diketiminate)_4_ complexes are even less common and typically require minimal steric bulk at nitrogen donors. These trends highlight the combined influence of steric congestion and electronic factors on limiting coordination number. More broadly, the analysis establishes that ligand modification provides a predictable means to tune redox properties and metal–ligand interactions, offering valuable design principles for applications in catalysis, materials chemistry, and bioinorganic modelling.

Collectively, these findings highlight that subtle donor atom substitution within a conserved chelate scaffold provides a powerful strategy for tuning ligand acidity, coordination strength, redox activity, and stability. The close structural analogy among β-diketones, enaminones, and β-diketiminates makes them ideal platforms for systematic structure–property investigations and rational ligand development.

## 4. Future Directions

Although β-diketones and their nitrogen analogues are well established in coordination chemistry, several avenues remain open for further exploration.

### 4.1. Rational Electronic Tuning Through Substituent Engineering

The demonstrated linear correlations between experimental reduction potentials and computed LUMO energies suggest that computationally guided ligand design can be expanded further. Systematic variation of electron-donating and electron-withdrawing substituents—particularly at positions directly conjugated with the chelate backbone—offers opportunities to precisely modulate redox potentials for applications in catalysis, electrocatalysis, and molecular electronics. Expanding these studies to include solvent effects and metal-bound systems would provide deeper insight into structure–property relationships under operational conditions.

### 4.2. Redox-Active and Non-Innocent Behaviour

The delocalized frontier orbitals and stabilized radical anions observed for enaminones and β-diketiminates indicate potential non-innocent behaviour when coordinated to redox-active metals. Future work could focus on ligand-centred versus metal-centred redox processes, particularly in catalytic cycles involving multi-electron transformations. Time-resolved spectroscopic and electrochemical techniques, combined with advanced DFT or multireference methods, may clarify charge distribution during redox events.

### 4.3. Advanced Materials and Thin-Film Precursors

β-Ketoiminate and related complexes have already shown promise as CVD precursors. Further development of fluorinated, heteroaryl, or perfluoroalkyl-substituted derivatives may enhance volatility, thermal stability, and decomposition control. Integration of these ligands into atomic layer deposition (ALD) processes and exploration of their behaviour under plasma-enhanced conditions represent promising research directions for materials chemistry.

### 4.4. Catalysis and Small-Molecule Activation

The strong σ-donor character of β-diketiminates, combined with steric tuneability, makes them attractive for stabilizing low-coordinate or low-valent metal centres. Expanding their application in small-molecule activation (e.g., CO_2_, N_2_O, or H_2_ activation) and sustainable catalytic processes is a logical extension. Comparative studies among O,O′-, N,O-, and N,N′-ligand systems within identical metal frameworks could provide valuable mechanistic insight into donor effects on catalytic performance.

### 4.5. Bioactive and Functional Molecules

Enaminones continue to attract interest in medicinal chemistry due to their hydrogen bonding capability and tuneable lipophilicity. Future interdisciplinary research linking coordination chemistry with biological evaluation may yield multifunctional metal–ligand systems with therapeutic or diagnostic potential.

In summary, β-diketones and their mono- and di-imine derivatives form a coherent and versatile family of chelating ligands. Their shared conjugated backbone, combined with systematic donor atom variation, provides a powerful framework for understanding and tailoring structural, electronic, and redox properties. Continued integration of experimental characterization with computational modelling is expected to further advance the rational design of functional ligand systems for applications spanning catalysis, materials science, and beyond.

## Data Availability

No new data are reported in this review.

## Abbreviations

The following abbreviations are used in this manuscript:

Hacac	2,4-pentanedione, acetylacetone
Hba	1-phenyl-1,3-butanedione, benzoylacetone
Hbfcm	1-ferrocenyl-3-phenylpropane-1,3-dione, benzoylferrocenoylmethane
Hbth	1-phenyl-3-(2-thenoyl)-1,3-propanedione
Hdbm	1,3-diphenyl-1,3-propanedione, dibenzoylmethane
Hdfcm	1,3-diferrocenylpropane-1,3-dione, diferrocenoylmethane
Hdpm	2,2,6,6-tetramethyl-3,5-heptanedione (dipivaloylmethane)
Hdtm	1,3-di(2-thenoyl)-1,3-propanedione
Hfca	1-ferrocenylbutane-1,3-dione, ferrocenoylacetone
Hfch	2-ferrocenoyl-etan-1-al, ferrocenoylaldehyde
Hfctca	1-ferrocenyl-4,4,4-trichlorobutane-1,3-dione, ferrocenoyltrichloroacetone
Hfctfa	1-ferrocenyl-4,4,4-trifluorobutane-1,3-dione, ferrocenoyltrifluoroacetone
Hhfaa	1,1,1,5,5,5-hexafluoro-2,4-pentanedione, hexafluoroacetylacetone
Htfaa	1,1,1-trifluoro-2,4-pentanedione, trifluoroacetylacetone
Htfba	1,1,1-trifluoro-4-phenyl-2,4-butanedione, trifluorobenzoylacetone
Htfdma	1,1,1-trifluoro-5-methyl-2,4-hexanedione
Htfhd	1,1,1-trifluoro-2,4-hexanedione
Htftma	1,1,1-trifluoro-5,5-dimethyl-2,4-hexanedione
Htta	thenoyltrifluoroacetone, 4,4,4-trifluoro-1-(2-thenoyl)-1,3-propanedione
